# Kinsenoside Targets IDH1 to Restore Microglial Immune‐Metabolic Homeostasis for Alzheimer's Disease Therapy

**DOI:** 10.1002/advs.75125

**Published:** 2026-04-14

**Authors:** Qianqian Li, Yajin Liao, Yan‐bo Zhao, Hongxing Wu, Tong Jin, Shuoshuo Li, Yuhan Liu, Peng Li, Songying Ouyang, Zekai Li, YuTing Xia, Qian Hua, Rui‐Yuan Pan, Zengqiang Yuan

**Affiliations:** ^1^ The Brain Science Center Beijing Institute of Basic Medical Sciences Beijing China; ^2^ Department of Neurology The Second Affiliated Hospital Hengyang Medical School University of South China Hengyang China; ^3^ NHC Key Laboratory of Neurodegenerative diseases (University of South China) Hengyang China; ^4^ Key Laboratory of Microbial Pathogenesis and Interventions of Fujian Province University the Key Laboratory of Innate Immune Biology of Fujian Province Biomedical Research Center of South China College of Life Sciences Fujian Normal University Fuzhou China; ^5^ Key Laboratory of Mental Health of the Ministry of Education Guangdong‐Hong Kong‐Macao Greater Bay Area Center for Brain Science and Brain‐Inspired Intelligence Guangdong‐Hong Kong Joint Laboratory for Psychiatric Disorders Guangdong Province Key Laboratory of Psychiatric Disorders Guangdong Basic Research Center of Excellence for Integrated Traditional and Western Medicine for Qingzhi Diseases Department of Neurobiology School of Basic Medical Sciences Southern Medical University Guangzhou China; ^6^ School of Life Sciences Beijing University of Chinese Medicine Beijing China; ^7^ School of Traditional Chinese Medicine Tianjin University of Traditional Chinese Medicine Tianjin China; ^8^ College of Basic Sciences College of Basic Sciences Shanxi Agricultural University Jinzhong China; ^9^ Key Laboratory of Clinical Neurology (Hebei Medical University) Ministry of Education Shijiazhuang Hebei China; ^10^ The Key Research Laboratory of Benefiting Qi for Acting Blood Circulation Method to Treat Multiple Sclerosis of State Administration of Traditional Chinese Medicine/Research Center of Neurobiology Shanxi University of Chinese Medicine Jinzhong China

**Keywords:** alzheimer's disease, kinsenoside, microglia, neuroinflammation, tca cycle, IDH1

## Abstract

Dysregulated tricarboxylic acid (TCA) cycle activity is increasingly recognized as a contributor to Alzheimer's disease (AD) pathogenesis, yet the mechanistic underpinnings of the relationship remain unclear. Here, we identify isocitrate dehydrogenase 1 (IDH1), a key enzyme in the TCA cycle, as a critical pathogenic driver of AD in microglia. IDH1 expression was markedly upregulated in microglia from both AD patients and 5×FAD mice. Elevated IDH1 promoted excessive cytosolic citrate consumption, which restricted citrate shuttling into mitochondria and impaired mitochondrial TCA cycle function. This citrate metabolic imbalance further disrupted epigenetic regulation, thereby exacerbating AD‐related pathological processes. Using structure‐based screening and co‐crystallization analysis, we identified Kinsenoside (KIN), a natural small molecule, as a selective competitive inhibitor of IDH1 that binds to its isocitrate‐binding pocket. Targeting IDH1 with KIN inhibited its activity, which restored intracellular citrate distribution, reactivated mitochondrial TCA cycle flux, and reestablished metabolic homeostasis. Notably, this intervention not only attenuated neuroinflammation but also reduced β‐amyloid (Aβ) deposition and significantly improved cognitive performance in 5×FAD mice. Collectively, our findings establish IDH1‐mediated metabolic dysregulation as a pivotal pathogenic mechanism in AD and highlight KIN as a promising therapeutic candidate by targeting microglial IDH1 to restore metabolic and functional homeostasis.

## Graphical abstract

Dysregulated TCA cycle contributes to Alzheimer's disease (AD) pathogenesis. Here, we show that microglial isocitrate dehydrogenase 1 (IDH1) is a critical driver. Elevated IDH1 disrupts citrate metabolism and mitochondrial function, exacerbating AD pathology. The natural small molecule kinsenoside (KIN) selectively inhibits IDH1, restoring metabolic homeostasis, reducing neuroinflammation and Aβ deposition, and improving cognitive function in 5×FAD mice.

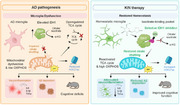

## 1 Introduction

Alzheimer's disease (AD), the most prevalent neurodegenerative disorder and leading cause of dementia worldwide, is characterized by progressive cognitive deterioration alongside its defining pathological hallmarks—amyloid‐beta (Aβ) plaques and neurofibrillary tangles [1, 2]. Despite decades of intensive research, disease‐modifying therapies with robust clinical efficacy remain elusive. This critical unmet need underscores the urgency of elucidating novel pathogenic mechanisms and identifying actionable therapeutic targets that govern AD progression [3, 4].

Emerging evidence increasingly implicates microglial metabolic dysregulation as a pivotal contributor to AD pathogenesis [5, 6]. As the primary resident immune cells of the central nervous system (CNS), microglia are indispensable for maintaining CNS homeostasis through core functions including immune surveillance, synaptic pruning, and clearance of cellular debris [7, 8]. However, in pathological settings like AD, microglia undergo profound metabolic reprogramming: a shift from mitochondrial oxidative phosphorylation (OXPHOS) to aerobic glycolysis that promotes chronic neuroinflammation and subsequent neuronal injury [9, 10, 11]. This maladaptive metabolic shift not only amplifies inflammatory responses but also impairs essential microglial effector functions, collectively highlighting that restoring microglial metabolic homeostasis represents a promising and actionable strategy for AD intervention.

The tricarboxylic acid (TCA) cycle stands as the cornerstone of cellular energy metabolism and metabolic homeostasis [12]. TCA cycle dysregulation is a well‐documented driver of mitochondrial dysfunction, cellular energy deficits, oxidative stress, and ultimately neuronal death [13, 14, 15]. Isocitrate dehydrogenase 1 (IDH1)—a key rate‐limiting enzyme in the TCA cycle—catalyzes the oxidative decarboxylation of isocitrate to α‐ketoglutarate (α‐KG), a pleiotropic metabolite that serves both as a critical energy substrate and as an essential cofactor for epigenetic regulators [16]. While IDH1 mutations have been extensively characterized in cancer, where they drive production of the oncometabolite 2‐hydroxyglutarate (2‐HG) and induce pathogenic epigenetic reprogramming [17, 18, 19], the functional role of wild‐type (WT) IDH1 in regulating microglial metabolism—and its potential relevance to AD pathogenesis—remains entirely unexplored. This critical knowledge gap represents a major barrier to fully elucidating how microglial metabolic dysregulation contributes to neurodegenerative processes in AD.

Herein, we define a novel pathogenic role of WT IDH1 in AD progression. We demonstrate that IDH1 is upregulated in microglia from both AD patients and 5×FAD mice, and this upregulation drives profound metabolic and inflammatory dysregulation in these cells. Elevated IDH1 levels reduce mitochondrial citrate availability, which disrupts TCA cycle flux and impairs ATP production—key hallmarks of metabolic dysfunction. Concurrently, cytosolic citrate metabolic imbalance induced by heightened IDH1 activity further perturbs epigenetic regulation, which in turn dysregulates microglial homeostasis and amplifies neuroinflammation.

To therapeutically target this IDH1‐mediated pathogenic axis, we performed a structure‐based virtual screen of 23 304 natural compounds, leading to the identification of KIN as a potent and selective inhibitor of WT IDH1. Biochemical and structural analyses confirmed KIN's specificity and efficacy, validating it as a promising AD therapeutic candidate. In vivo studies further demonstrated that KIN treatment reactivates mitochondrial metabolism, dampens proinflammatory signaling cascades, and reduces Aβ plaque burden in 5×FAD mice—effects that collectively rescue cognitive deficits. Our findings establish IDH1 as a critical metabolic switch that integrates cellular metabolism, epigenetic remodeling, and immune activation in AD, thereby providing a robust mechanistic foundation for the development of IDH1‐targeted disease‐modifying interventions.

## 2 Results

### 2.1 IDH1 Expression and Activity are Elevated in AD‐Associated Microglia

**FIGURE 1:**
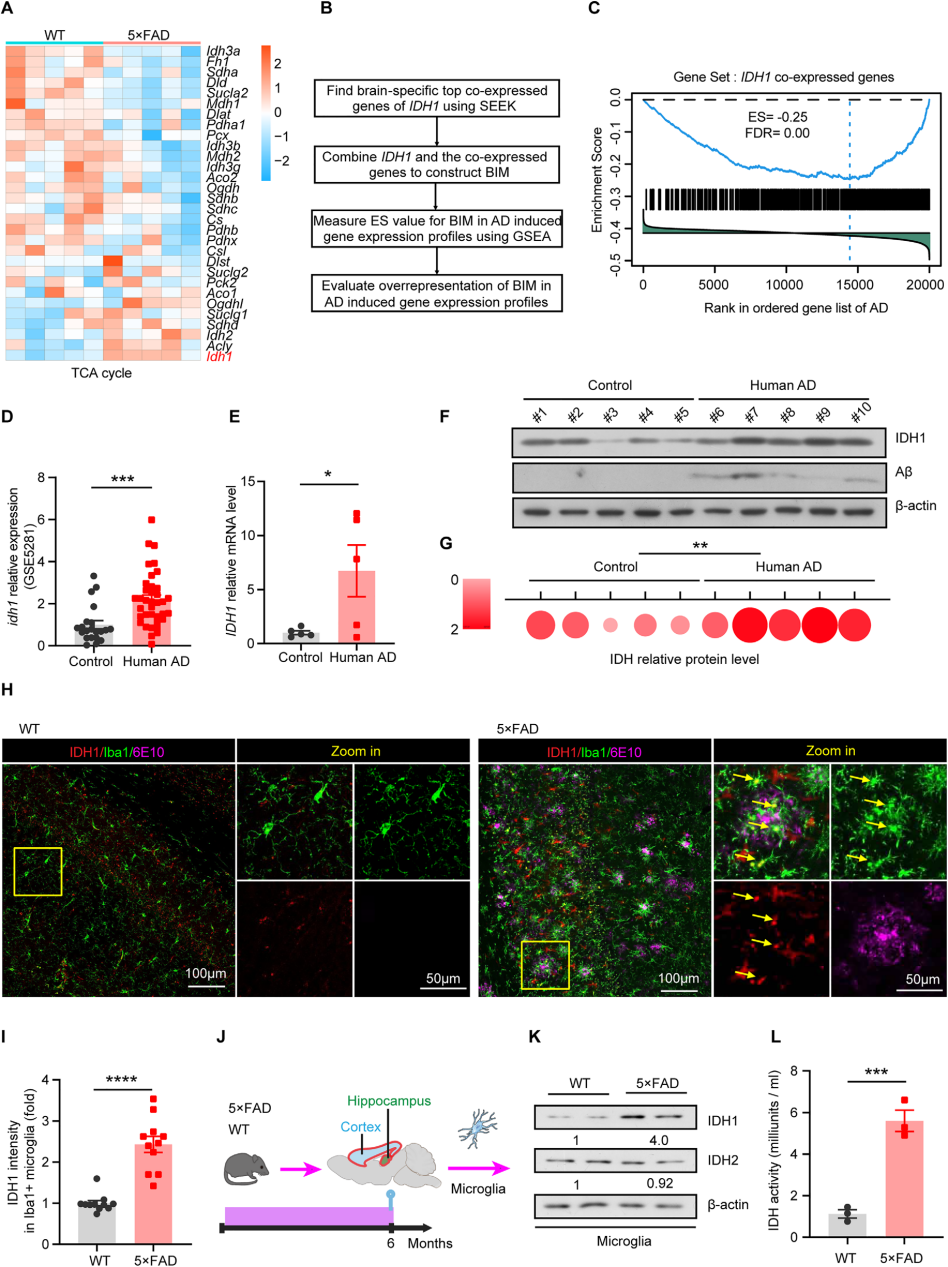
IDH1 expression is elevated in microglia from AD patients and 5×FAD mice. (A) Heatmap showing the relative mRNA expression levels of enzymes involved in the tricarboxylic acid (TCA) cycle in brain tissues from WT mice and 5×FAD mice. (B) Transcriptome‐based functional gene module reference method to evaluate the enrichment of the brain‐specific *IDH1* co‐expressed gene module (BIM) in AD induced gene expression profiles. (C) Gene set enrichment analysis (GSEA) plot depicting the enrichment of *IDH1* co‐expressed genes in the transcriptional profile of AD. (D) *IDH1* mRNA levels in brain tissues from patients with AD (*n* = 37) and age‐matched healthy individuals (*n* = 21), analyzed using the GEO dataset GSE5281. (E) *IDH1* mRNA expression in postmortem brain sections of patients with AD and healthy controls (*n* = 5 per group). (F) Western blotting analysis of IDH1 protein levels in the frontal lobes of patients with AD and healthy controls (*n* = 5 per group). (G) Quantification of IDH1 protein expression from panel (F) (*n* = 5 per group). (H) Representative immunofluorescence images of IDH1 and Iba1 co‐staining in brains of 6‐month‐old WT and 5×FAD mice. Yellow arrowheads indicate microglia adjacent to plaques with elevated IDH1 expression. (I) Quantification of IDH1 fluorescence intensity in Iba1^+^ microglia from panel (H). (J) Schematic illustrating the experimental design of microglia isolated from 5×FAD mice or vehicle control. (K) Western blotting analysis of IDH1 in microglia isolated from 6‐month‐old WT and 5×FAD mice. (L) Measurement of IDH1 enzymatic activity in microglia isolated from 6‐month‐old WT and 5×FAD mice. Data are presented as mean ± SEM. ^*^ *p* < 0.05, ^**^ *p* < 0.01, ^***^ *p* < 0.001, ^****^ *p* < 0.0001. Statistical comparisons were performed using a two‐tailed unpaired Student's t‐test (panels A, D, E, G, I, and L).

To investigate the potential involvement of TCA cycle enzymes in AD pathogenesis, we first analyzed the transcriptomes of brain tissues from WT mice and 5×FAD mice. Quantitative analysis of these datasets revealed that among all TCA cycle enzymes, *Idh1* mRNA levels were significantly higher in the brains of 5×FAD mice compared to WT controls (Figure 1A). To investigate the link between IDH1 and AD, we employed a transcriptome‐based functional gene module reference approach (Figure 1B) [20, 21], which allowed us to both examine their relationship and further dissect their functional interplay. Briefly, we constructed a brain‐specific IDH1 co‐expression module (BIM) by integrating IDH1 with its top co‐expressed genes in brain tissues (Table S1). We then performed gene set enrichment analysis (GSEA) to characterize BIM enrichment in AD transcriptional profiles. From the Gene Expression Omnibus (GEO) database, we curated AD transcriptional datasets spanning multiple key brain regions affected by AD pathology: hippocampus, temporal cortex, and frontal cortex. BIM genes were significantly downregulated in the gene expression profiles of all three regions (Table S2). Notably, the hippocampus exhibited the highest BIM enrichment score, consistent with its role as a primary site of AD‐related pathological damage (Figure 1C). Collectively, these results confirm a functional association between the IDH1 co‐expression module and AD.

To extend these findings and validate the relevance of IDH1 dysregulation to AD, we systematically examined IDH1 expression in both human tissue and mouse models. Analysis of GEO datasets (GSE5281 and GSE28146) revealed significantly elevated *IDH1* mRNA levels in brain tissues of AD patients compared to healthy controls (Figure 1D; Figure S1A). These transcriptomic observations were corroborated by quantitative PCR (qPCR) analysis of postmortem brain specimens, which confirmed increased *IDH1* mRNA expression in AD patients (Figure 1E). Western blotting further validated this dysregulation at the protein level, demonstrating heightened IDH1 abundance in the frontal lobes of AD brains (Figure 1F,G). Notably, mRNA expression levels of other IDH isoforms remained unaltered (Figure S1B‐G), highlighting the specificity of IDH1 dysregulation in AD.

We next delineated the cellular localization of IDH1 in AD by performing immunofluorescence staining on brain tissue sections from 5×FAD mice, co‐labeling IDH1, the microglial marker Iba1, and Aβ plaques (via the 6E10 antibody). IDH1 immunoreactivity was significantly stronger in plaque‐associated microglia of 5×FAD mice relative to age‐matched WT littermates (Figure 1H,I). Western blotting of proteins extracted from isolated microglia of 5×FAD mice further confirmed this upregulation at the protein level (Figure 1J,K). Consistent with increased protein expression, enzymatic activity assays demonstrated enhanced IDH1 catalytic activity in microglia from AD mice compared to WT controls (Figure 1L). Collectively, these data establish that both the expression and enzymatic activity of IDH1 are specifically and significantly elevated in AD‐associated microglia, pinpointing microglial IDH1 as a dysregulated mediator in AD pathogenesis.

### 2.2 Microglial Idh1 Deletion Ameliorates Cognitive Deficits, Aβ Pathology, and Neuroinflammation in 5×FAD Mice

**FIGURE 2:**
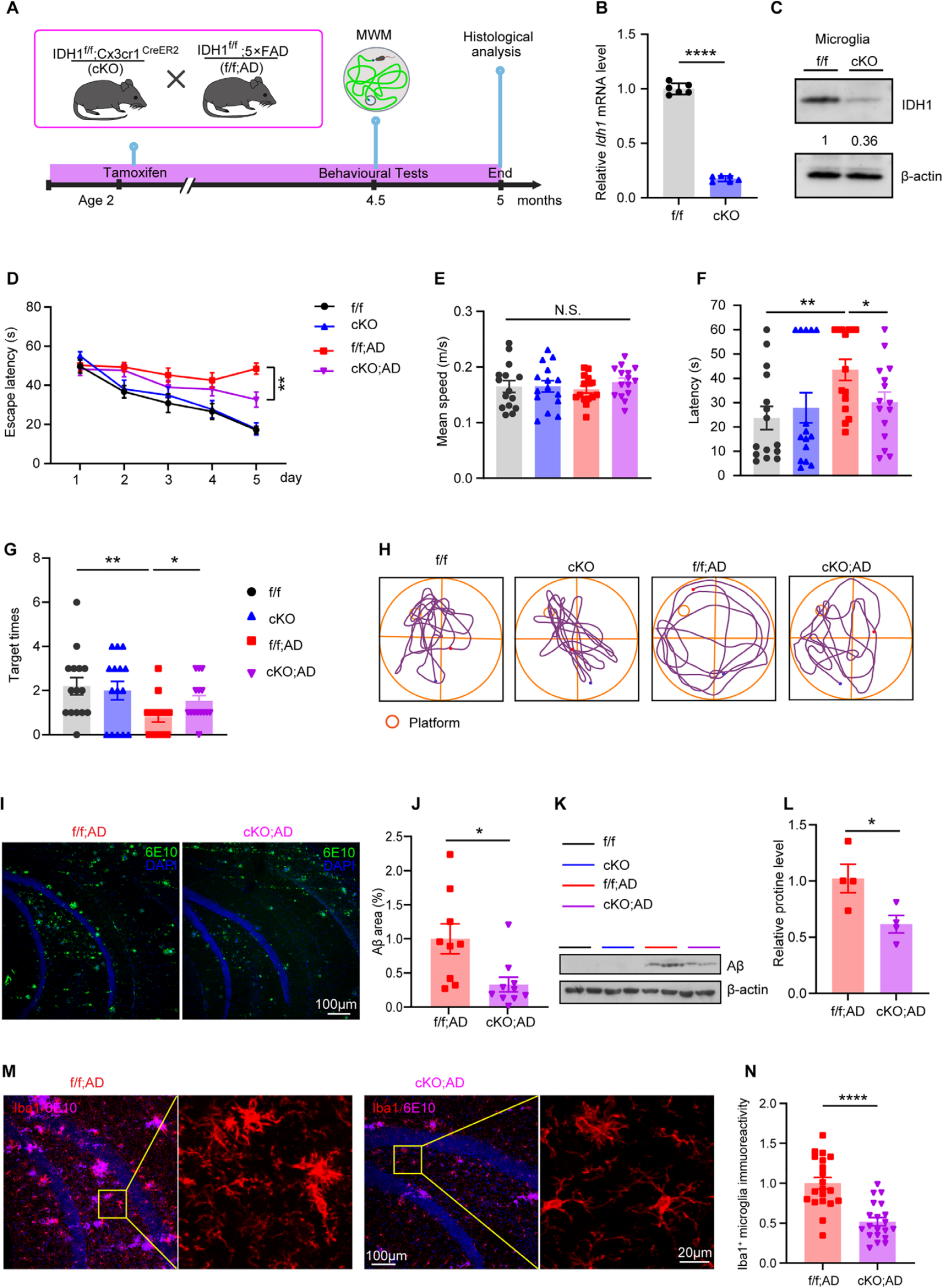
Microglial *IDH1* ablation alleviates cognitive deficits and Aβ pathology in 5×FAD mice. (A) Schematic of the strategy used to delete *Idh1* in microglia of 5×FAD mice. (B) Quantitative analysis of relative *Idh1* mRNA levels in microglia of f/f control mice and cKO mice. (C) Western blotting confirming *Idh1* deletion efficiency in microglia, representative of three independent experiments. (D) Escape latency during Morris water maze training trials (*n* = 15 mice per group: f/f, cKO, f/f;AD, and cKO;AD). (E–G) Quantification of mean swimming speed (E), latency to first entry into the target quadrant (F), and number of target entries (G) during the probe trial (*n* = 15 per group). (H) Representative swimming tracks during the probe trial. (I) Representative 6E10 immunostaining of Aβ plaques in brain sections of f/f;AD and cKO;AD mice. (J) Quantification of 6E10‐positive Aβ plaque area in the dentate gyrus of 5‐month‐old f/f;AD and cKO;AD mice (*n* = 4 per group). (K–L) Western blotting analysis of Aβ in whole brain lysates from 5‐month‐old f/f, cKO, f/f;AD, and cKO;AD mice (K), with corresponding quantification (L) (*n* = 4 per group). (M) Representative images of microglia (Iba1) in the hippocampal DG region of 5‐month‐old f/f;AD and cKO;AD mice (*n* = 4 per group). (N) Quantification of Iba1^+^ microglial immunoreactivity in the DG region (*n* = 4 per group). Data are presented as mean ± SEM. ^*^ *p* < 0.05, ^**^ *p* < 0.01, ^****^ *p* < 0.0001; N.S., not significant. Statistical comparisons were performed using two‐tailed unpaired Student's *t*‐test (B, J, L, N) or one‐way (E–G) or two‐way ANOVA followed by Tukey's multiple comparisons test (D).

To delineate the functional role of microglial IDH1 in AD pathogenesis, we generated microglia‐specific *Idh1* knockout mice by crossing *Idh1* ^f/f^ mice with *Cx3cr1* ^CreERT2^ and 5×FAD mice (Figure 2A), resulting in four genotypes: f/f (*Idh1* ^f/f^), f/f;cKO (*Idh1* ^f/f^;*Cx3cr1* ^CreERT2^), f/f;AD (*Idh1* ^f/f^;5×FAD), and cKO;AD (*Idh1* ^f/f^;*Cx3cr1* ^CreERT2^). We first examined *Idh1* transcript abundance in microglia, which demonstrated a dramatic reduction in *Idh1* mRNA levels in f/f;cKO mice relative to f/f control mice (Figure 2B). Western blotting of isolated microglia further confirmed efficient depletion of IDH1 protein in the cKO group (Figure 2C). Following this molecular characterization, we first assessed cognitive function using the Morris water maze test. Compared to f/f;AD controls, cKO;AD mice exhibited significantly shorter latencies to locate the hidden platform during training trials (Figure 2D). In probe trials, cKO;AD mice also showed more frequent entries into the platform zone and increased time spent in the target quadrant (Figure 2E‐H)—collectively indicating improved spatial learning and memory. Notably, swimming speeds were comparable across all groups, excluding motor deficits as a confounding factor (Figure 2E).

We next investigated the impact of microglial *Idh1* deletion on Aβ pathology using immunofluorescence staining and Western blotting with the Aβ‐specific antibody 6E10. Immunofluorescence analysis revealed a marked reduction in the area occupied by Aβ plaques in the hippocampal dentate gyrus of cKO;AD mice compared to f/f;AD littermates (Figure 2I,J). These histological findings were corroborated by Western blotting, which demonstrated significantly decreased Aβ protein levels in the hippocampus of cKO;AD mice (Figure 2K–L).

Neuroinflammation is a central contributor to AD pathogenesis [22], and our previous work has linked microglial metabolic dysregulation to exacerbated neuroinflammatory responses and accelerated AD pathogenesis [11]. To evaluate the effect of microglial Idh1 deletion on microglial activation and neuroinflammation, we performed immunostaining for the microglial marker Iba1 in brain sections from 5×FAD mice. Compared to f/f;AD mice, cKO;AD mice displayed a significant reduction in the number of Iba1^+^ microglia in the hippocampal dentate gyrus—indicating attenuated microglial activation (Figure 2M,N).

To validate this anti‐inflammatory effect, we knocked down *Idh1* in primary cultured microglia using small interfering RNA (siRNA). In Aβ‐stimulated microglia, *Idh1* knockdown markedly suppressed the expression of the pro‐inflammatory cytokines *Il‐6*, *Il‐1β*, and *Tnf‐α* at both 3‐ and 6‐ h post‐stimulation (Figure S2A–D). Collectively, these results demonstrate that ablation of *Idh1* in microglia mitigates Aβ pathology and AD‐associated neuroinflammation while rescuing cognitive deficits in 5×FAD mice—underscoring a critical pathogenic role of microglial IDH1 in AD progression.

### 2.3 IDH1 Deficiency Reactivates the TCA Cycle and Restores Energetic Metabolism in AD‐Associated Microglia

**FIGURE 3:**
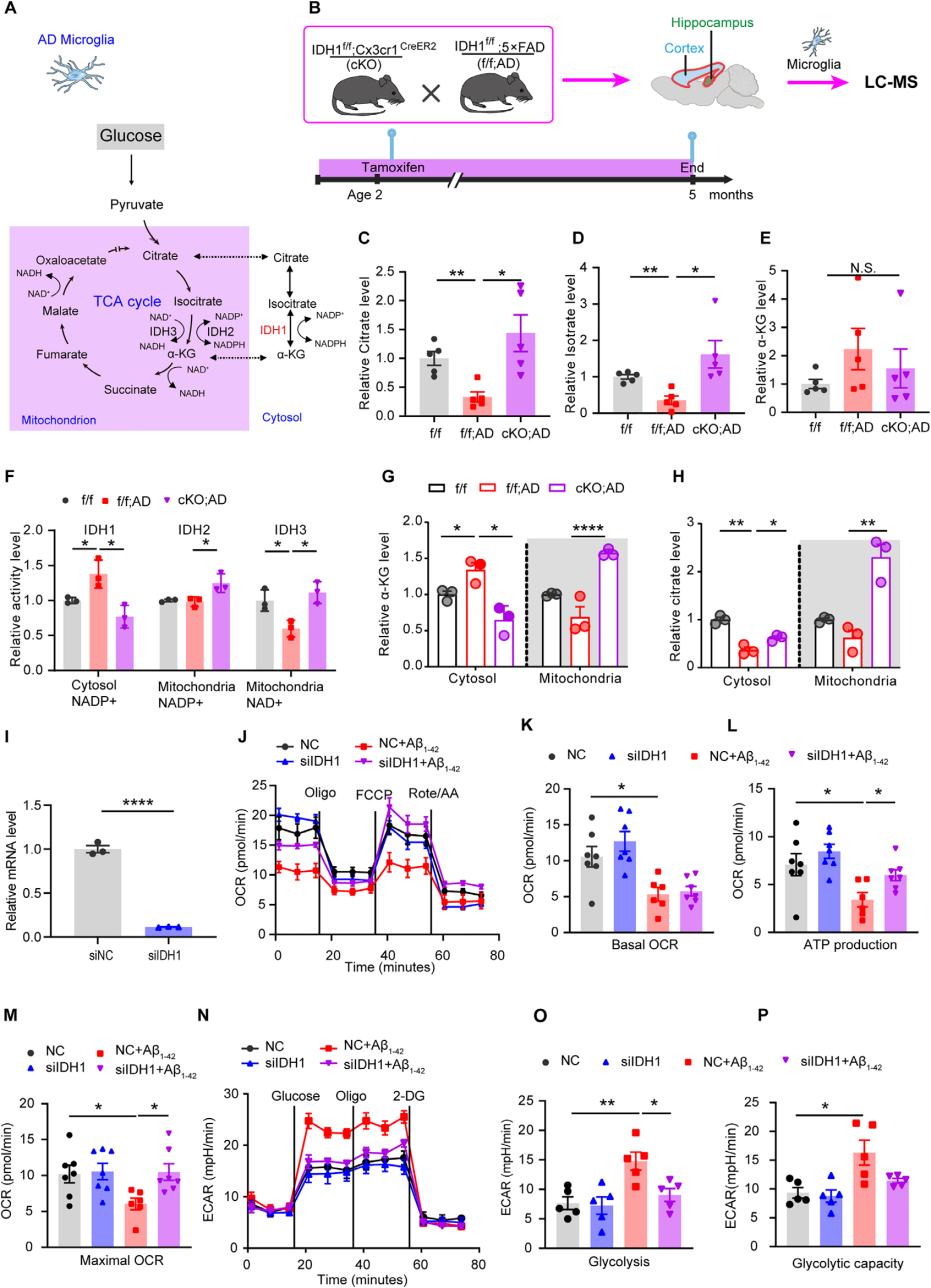
*IDH1* deficiency enhances microglial TCA cycle activity and energy metabolism in 5×FAD mice. (A) Schematic of IDH‐associated metabolic pathways. (B) Schematic illustrating the experimental design for untargeted LC‐MS‐based metabolomic profiling of microglia isolated from 5‐month‐old f/f, f/f;AD, and cKO;AD mice. (C–E) Relative quantification of citrate, isocitrate, and α‐KG in microglia from 5‐month‐old f/f, f/f;AD, and cKO;AD mice. (F) IDH enzymatic activity in microglial cytosolic and mitochondrial fractions using NADP^+^ (IDH1/2) or NAD^+^ (IDH3) as cofactors across f/f, f/f;AD, and cKO;AD mice. (G–H) Quantification of citrate (G) and α‐KG (H) levels in microglial mitochondrial and cytosolic compartments from 5‐month‐old f/f, f/f;AD, and cKO;AD mice. (I) *Idh1* mRNA expression in primary microglia transfected with *siNC* or *siIDH1*, measured by qPCR. (J–M) OCR analysis of primary microglia exposed to Aβ following *siNC* or *siIDH1* transfection (J), with quantification of basal OCR (K), ATP production (L), and maximal OCR (M) (n = 5 replicate wells). (N–P) ECAR analysis of primary microglia exposed to Aβ following siNC or siIDH1 transfection for 24 h (N), with quantification of glycolysis (O), and glycolytic capacity (P) (n = 5 replicate wells). Data are presented as mean ± SEM. ^*^ *p* < 0.05, ^**^ *p* < 0.01, ^****^ *p* < 0.0001; N.S., not significant. Statistical comparisons were made using two‐tailed unpaired Student's *t*‐test (I) or one‐way ANOVA followed by Tukey's multiple comparisons test (C–H, K–M, and O–P).

IDH isoenzymes catalyze the oxidative decarboxylation of isocitrate to α‐KG—a rate‐limiting step in the TCA cycle that is tightly coupled to cellular redox homeostasis. Among IDH isoforms, mitochondrial NAD^+^‐dependent IDH3 is the primary driver of TCA cycle flux, whereas cytosolic NADP^+^‐dependent IDH1 and mitochondrial NADP^+^‐dependent IDH2 contribute to NADPH generation and antioxidant defense (Figure 3A). To delineate the metabolic impact of IDH1 dysregulation in AD microglia, we performed metabolomic profiling of microglia from 5‐month‐old f/f, f/f;AD, and cKO;AD mice. Mass spectrometry (MS)‐based metabolite quantification revealed that elevated IDH1 expression in f/f;AD microglia correlated with reduced intracellular citrate and isocitrate levels—deficits that were fully restored upon *Idh1* deletion. Notably, total cellular α‐KG levels remained unchanged across groups (Figure 3B–E). Enzymatic activity assays further uncovered compartment‐specific dysregulation of IDH isoforms in AD microglia: f/f;AD microglia exhibited decreased mitochondrial IDH3 activity alongside elevated cytosolic IDH1 activity. This isoenzyme imbalance was reversed in cKO;AD microglia, which displayed increased mitochondrial IDH2/3 activity and diminished cytosolic IDH1 activity (Figure 3F).

To dissect the metabolic consequences of this compartmentalized dysregulation, we measured citrate and α‐KG levels in isolated mitochondrial and cytosolic fractions. In f/f;AD microglia, mitochondrial citrate and α‐KG pools were depleted, while cytosolic citrate levels were reduced and cytosolic α‐KG was elevated—metabolic perturbations that were normalized in cKO;AD microglia (Figure 3G,H). These findings indicate that Idh1 deletion restores mitochondrial citrate availability and reactivates TCA cycle flux in AD microglia. Beyond its role in mitochondrial metabolism, cytosolic citrate, which freely shuttles between the cytosol and nucleus—is catabolized by ATP‐citrate lyase (ACLY) to generate acetyl‐CoA, a critical metabolite that links cellular metabolism to transcriptional regulation via histone acetylation [21]. A growing body of evidence demonstrates that increased levels of histone H3 lysine 27 acetylation (H3K27ac) ameliorate AD‐like pathologies and improve cognitive function in multiple AD mouse models [23, 24, 25, 26]. Consistent with this, we found that Idh1 depletion in microglia significantly elevated H3K27ac levels (Figure S3A,B), supporting a model where IDH1 regulates AD pathology via metabolism‐epigenetics crosstalk.

To directly assess the impact of IDH1 on mitochondrial bioenergetic function, we measured extracellular acidification rate (ECAR) and oxygen consumption rate (OCR) in primary microglia using a Seahorse XF 96 Extracellular Flux Analyzer. Aβ exposure suppressed basal OCR, ATP‐linked respiration, and maximal respiratory capacity, while concurrently enhancing glycolytic capacity and suppressing glycolysis—metabolic defects that were reversed by *Idh1* knockdown (Figure 3I–P). These data confirm that *Idh1* ablation induces metabolic reprogramming of AD microglia from a glycolytic phenotype back to OXPHOS dependence. Collectively, these findings establish that microglial Idh1 deletion restores mitochondrial TCA cycle activity, enhances OXPHOS, and mitigates metabolism‐linked epigenetic dysregulation—key perturbations associated with AD pathology.

### 2.4 KIN Identified as a Selective, High‐Affinity Inhibitor of Wild‐Type IDH1

**FIGURE 4:**
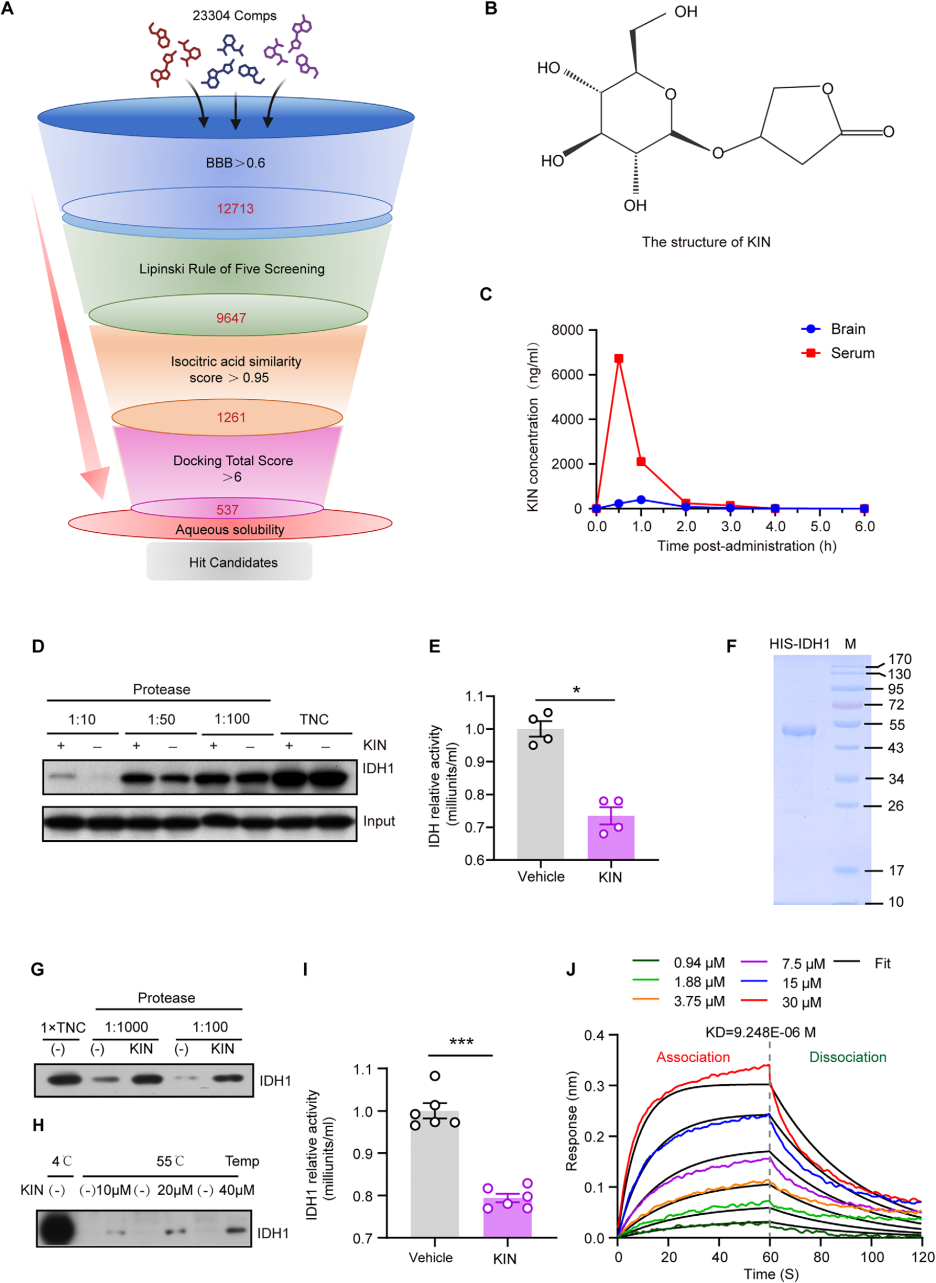
KIN is a selective inhibitor of IDH1. (A) Workflow for multi‐step virtual screening of 23,304 naturally occurring small molecules to identify potent IDH1 inhibitors. (B) Chemical structure of KIN. (C) Pharmacokinetics of KIN showing time‐dependent concentrations in the brain and serum. (D) DARTS‐based Western blotting analysis of KIN binding to IDH1 in primary cultured microglia. (E) IDH1 enzymatic activity in primary microglia treated with KIN or vehicle control. (F) Immunoblot analysis of recombinant HIS‐tagged IDH1. (G) DARTS Western blotting analysis of KIN interaction with recombinant HIS‐tagged IDH1. (H) CETSA Western blotting exhibits the binding affinity of HIS‐IDH1 to KIN. (I) Enzymatic activity of HIS‐IDH1 protein treated with KIN or vehicle control. (J) BLI measurement of KIN binding affinity (dissociation constant) to WT IDH1. Data are presented as mean ± SEM. ^*^ *p* < 0.05, ^***^ *p* < 0.001. Statistical comparisons were made using a two‐tailed unpaired Student's *t*‐test (E, I).

Given the established pathogenic role of IDH1 in AD‐associated microglia, we performed a multi‐step virtual screen of 23 304 small molecules to identify selective WT IDH1 inhibitors (Figure 4A). Screening criteria were stringently defined to prioritize drug‐like candidates: blood‐brain barrier (BBB) permeability (BBB score > 0.6), compliance with Lipinski's Rules (drug‐likeness), structural similarity to isocitrate (cosine similarity > 0.95), high docking affinity (docking score > 6), and favorable solubility profiles (Figure S4A–E). Application of these criteria identified KIN as a top lead compound (Figure 4B). Pharmacokinetic profiling demonstrated that KIN exhibits BBB penetration, achieving detectable brain levels following systemic administration (Figure 4C). To validate direct binding between KIN and IDH1, we performed drug affinity responsive target stability (DARTS) assays. Microglial lysates incubated with KIN retained significantly higher levels of IDH1 protein compared to vehicle‐treated controls, confirming a direct KIN‐IDH1 interaction (Figure 4D). Consistent with this binding, KIN treatment markedly inhibited IDH1 enzymatic activity in primary microglia (Figure 4E). Using recombinant His‐tagged WT IDH1, we further confirmed that KIN protects IDH1 from both proteolytic degradation and thermal denaturation, while concurrently suppressing its catalytic activity in vitro (Figure 4F–I). Bio‐layer interferometry (BLI) assays quantitatively demonstrated dose‐dependent binding of KIN to WT IDH1, with a dissociation constant (K) indicative of high‐affinity interaction (Figure 4J). Collectively, these biochemical and biophysical data establish KIN as a potent and selective inhibitor of WT IDH1.

**FIGURE 5:**
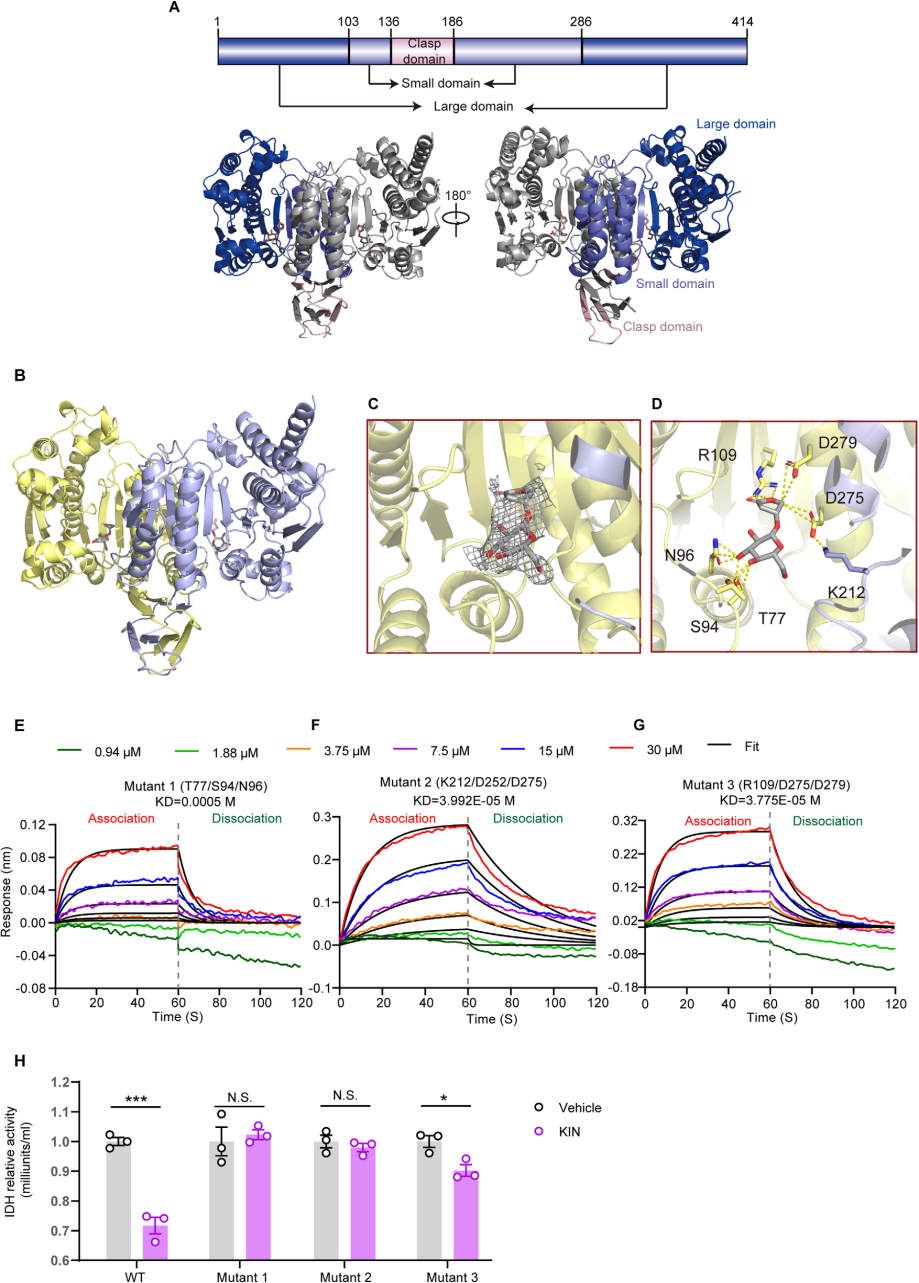
Identification and validation of KIN as a selective IDH1 inhibitor. (A) Domain architecture of IDH1: schematic representation of the large (blue), small (slate), and clasp (light pink) domains; 3D structure views are shown with 180° rotation to visualize spatial organization. (B) Crystal structure of the IDH1‐KIN complex, with the two IDH1 monomers shown in slate and yellow, and KIN depicted as a grey stick model. (C) Electron density map visualizing ligand KIN. (D) 3D interaction diagram of KIN and surrounding amino acid residues, with key residues depicted in stick format and hydrogen bonds shown as yellow dashed lines. (E–G) BLI measurements of KIN dissociation constants for mutant HIS‐IDH1 proteins. (H) Enzymatic activity of mutant HIS‐IDH1 protein treated with KIN or vehicle control. Data are presented as mean ± SEM. ^*^ *p* < 0.05, ^***^ *p* < 0.001, N.S., not significant. Statistical comparisons were made using a two‐tailed unpaired Student's *t*‐test (H).

To elucidate the molecular mechanism underlying KIN‐mediated IDH1 inhibition, we determined the crystal structure of the IDH1–KIN complex via single‐crystal X‐ray diffraction (refinement statistics provided in Table S3) (Figure 5A). The overall conformation of IDH1 in the complex was analogous to the previously reported IDH1–isocitrate (ICT)–Mn^2^^+^ complex (PDB: 1LWD); however, the IDH1–KIN structure lacked metal ion coordination—a key distinction from the substrate‐bound state. Instead, IDH1 residues D275 and D279, in conjunction with R109, formed hydrogen bonds with the aglycone moiety of KIN. Additionally, hydroxyl groups at the C‐3 and C‐4 positions of KIN's sugar ring engaged in hydrogen bonding with IDH1 residues T77, S94, and N96—all critical components of the substrate‐binding pocket (Figure 5B–D). Site‐directed mutagenesis of key residues within the IDH1 binding pocket abrogated KIN binding (Figure 5E–H). Taken together, these structural and mutational analyses demonstrate that KIN exerts selective, high‐affinity inhibition of IDH1 through direct binding to its substrate pocket and subsequent allosteric modulation of enzymatic activity.

### 2.5 KIN Reprograms Energetic Metabolism and Restores TCA Cycle Function in Microglia of 5×FAD Mice

**FIGURE 6:**
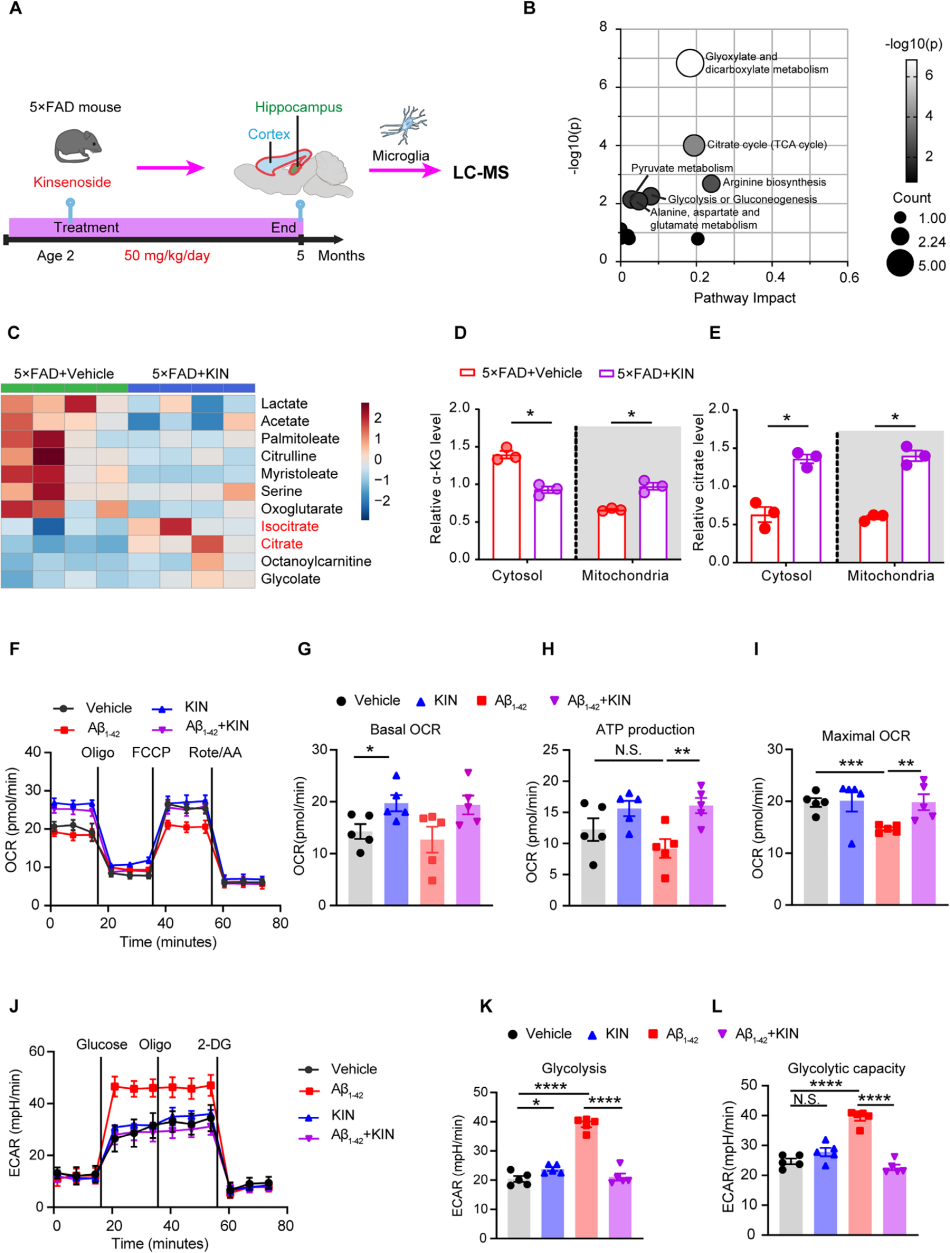
KIN enhances TCA cycle activity and energetic metabolism in microglia of 5×FAD mice. (A) Schematic illustrating the experimental design for untargeted LC‐MS‐based metabolomic profiling of microglia isolated from 5×FAD mice treated with KIN or vehicle control. (B) KEGG pathway enrichment analysis of differentially abundant metabolites in microglia following KIN treatment. (C) Untargeted LC‐MS metabolomics revealed differential metabolite profiles in microglia from 5×FAD mice treated with KIN vs. vehicle control. (D–E) Quantification of α‐KG (D) and citrate (E) in mitochondrial and cytosolic fractions of microglia isolated from 5×FAD mice treated with KIN or vehicle control (*n* = 3 per group). (F–I) Seahorse XF analysis of OCR in primary microglia exposed to Aβ and treated with 20 µM KIN or vehicle control for 24 h. Quantification includes basal OCR (G), ATP production (H), and maximal OCR (I) (n = 5 per group). (J–L) Seahorse XF analysis of ECAR in primary microglia exposed to Aβ and treated with 20 µm KIN or vehicle control for 24 h. Quantification includes glycolysis (K), and glycolytic capacity (L) (n = 5 replicate wells). Data are presented as mean ± SEM. ^*^ *p* < 0.05, ^**^ *p* < 0.01, ^***^ *p* < 0.001, ^****^ *p* < 0.0001; N.S., not significant. Statistical comparisons were made using two‐tailed unpaired Student's *t‐*test (D, E) or one‐way ANOVA (G–I, K–L) with Tukey's multiple comparisons test.

To investigate the impact of KIN on microglial energy metabolism in AD, we performed untargeted LC‐MS‐based metabolomic profiling of microglia isolated from 5‐month‐old 5×FAD mice treated with KIN or vehicle control (Figure 6A). Kyoto Encyclopedia of Genes and Genomes (KEGG) pathway analysis revealed extensive metabolic reprogramming in KIN‐treated microglia, with the TCA cycle being one of the most significantly affected pathways (Figure 6B). At the individual metabolite level, KIN treatment induced a marked increase in intracellular isocitrate and citrate levels, accompanied by a notable reduction in lactate (Figure 6C)—a metabolic signature indicative of a shift from aerobic glycolysis toward OXPHOS.

To delineate the subcellular specificity of these metabolic changes, we fractionated isolated microglia into cytosolic and mitochondrial compartments and quantified metabolite concentrations. KIN‐treated microglia exhibited significantly elevated mitochondrial levels of α‐KG and citrate, whereas cytosolic α‐KG levels were reduced relative to vehicle controls (Figure 6D,E). These findings suggest that KIN enhances mitochondrial citrate availability, thereby restoring TCA cycle activity in AD‐associated microglia. We next assessed the epigenetic consequences of KIN‐mediated IDH1 inhibition. Immunofluorescence staining demonstrated that KIN‐treated 5×FAD microglia—similar to cKO;AD microglia—exhibited significantly increased levels of histone H3K27ac compared to controls (Figure S5A,B), consistent with a model where metabolic restoration modulates histone acetylation status.

To further validate the effect of KIN on microglial bioenergetic function, we measured ECAR and OCR in primary microglia exposed to Aβ using a Seahorse extracellular flux analyzer. Aβ exposure markedly suppressed basal OCR, ATP‐linked respiration, maximal respiratory capacity, and glycolysis, while concurrently enhancing glycolytic capacity—all of these bioenergetic deficits were fully rescued by KIN treatment (Figure 6F–L). Collectively, these results establish that pharmacological inhibition of IDH1 by KIN reprograms microglial metabolism in the AD context, reactivating TCA cycle flux, promoting OXPHOS, and reversing metabolism‐linked epigenetic dysregulation.

### 2.6 KIN Improves Cognitive Function and Mitigates Aβ Pathology in 5×FAD Mice

**FIGURE 7:**
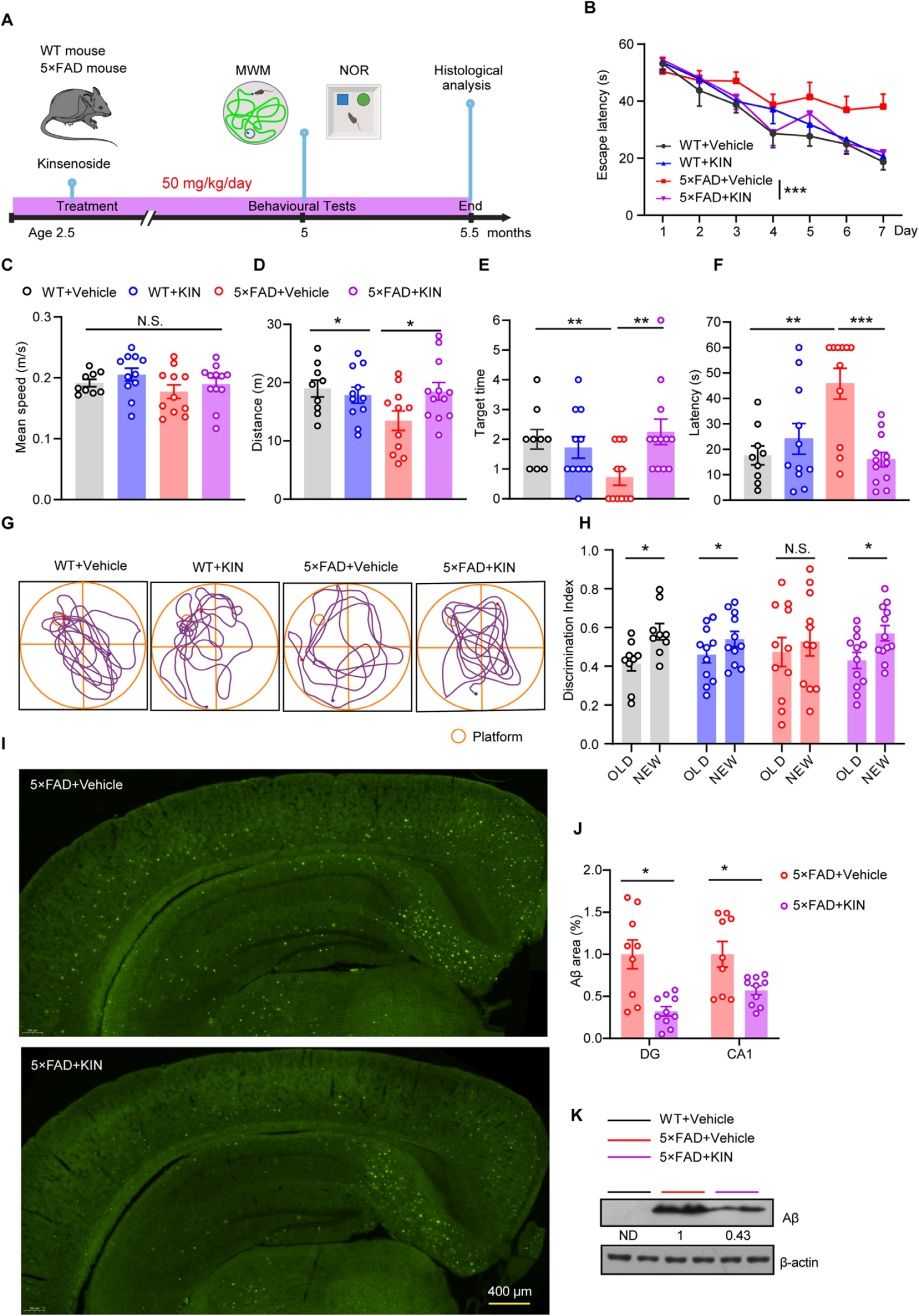
KIN ameliorates cognitive deficits and reduces Aβ burden in 5×FAD mice. (A) Schematic of KIN administration protocol in 5×FAD mice. (B) Escape latency during Morris water maze training trials (*n* = 9, 11, 11, and 12 for WT+Vehicle, WT+KIN, 5×FAD+Vehicle, and 5×FAD+KIN groups, respectively). (C) Average swim speed during the probe trial of the Morris water maze. (D–F) Performance metrics during the probe trial: distance traveled in the target quadrant (D), number of target entries (E), and latency to first target entry (F) (*n* = 9, 11, 11, and 12 per group, respectively). (G) Representative swim path traces during the probe trial. (H) Quantification of exploration time in the novel object recognition test (*n* = 9, 11, 11, and 12 per group). (I) Representative images of TS staining for Aβ plaques in brain sections of 5×FAD mice treated with KIN or vehicle control. (J) Quantification of TS‐stained Aβ plaque number and area in the dentate gyrus (DG) and CA1 regions. (K) Western blotting analysis of Aβ levels in brain homogenates from WT and 5×FAD mice treated with KIN or vehicle control (*n* = 4 mice per group). Data are presented as mean ± SEM. ^*^ *p* < 0.05, ^**^ *p* < 0.01, ^***^ *p* < 0.001; N.S., not significant. Statistical comparisons were made using two‐tailed unpaired Student's *t*‐test (H, J) or one‐way (C–F) or two‐way ANOVA followed by Tukey's multiple comparisons test (B).

To evaluate the in vivo intervention potential of KIN for AD, 2.5‐month‐old 5×FAD mice were administered KIN (50 mg/kg) via daily gavage for 10 weeks, followed by a comprehensive battery of behavioral tests and pathological analyses (Figure 7A). In the Morris water maze, during training trials, KIN‐treated 5×FAD mice exhibited significantly reduced escape latencies compared to vehicle‐treated controls, indicating improved spatial learning ability (Figure 7B). In subsequent probe trials, KIN‐treated mice displayed shorter latency to the former platform location, increased number of platform crossings, and prolonged time spent in the target quadrant (Figure 7C–G). Notably, swimming speeds were comparable across groups, excluding motor function deficits as a confounding factor (Figure 7C). These findings collectively confirm enhanced spatial memory retention in KIN‐treated 5×FAD mice. Parallel results in a 6‐month‐old 5×FAD mice showed that KIN intervention (50 mg/kg) similarly improved spatial learning and memory in behavioral tests (Figure S6A–G). To validate the cognitive benefits of KIN, we performed the novel object recognition (NOR) assay—a sensitive measure of recognition memory. Consistent with the Morris water maze results, KIN‐treated 5×FAD mice spent significantly more time exploring the novel object relative to the familiar one, confirming improved cognitive performance (Figure 7H).

We next assessed the impact of KIN on Aβ pathology, a defining hallmark of AD, using Thioflavin S (TS) staining—a gold‐standard method for detecting fibrillar Aβ plaques. Quantitative analysis revealed a marked reduction in Aβ plaque burden in the brains of KIN‐treated 5×FAD mice compared to vehicle controls (Figure 7I,J). These histological findings were corroborated by Western blotting, which demonstrated significantly decreased Aβ protein levels in the hippocampus of KIN‐treated mice (Figure 7K). Consistently, in the 6‐month‐old 5×FAD cohort, KIN treatment similarly reduced Aβ plaque burden in the brain (Figure S6H,I). Taken together, these in vivo data establish that KIN treatment mitigates cognitive impairments and reduces Aβ accumulation in 5×FAD mice, supporting its potential as a disease‐modifying therapeutic agent for AD.

### 2.7 KIN Attenuates Neuroinflammation by Suppressing Microglial Activation in 5×FAD Mice

Given our prior observation that microglial *Idh1* knockout potently suppresses neuroinflammation in 5×FAD mice, we next investigated whether KIN exerts comparable anti‐inflammatory effects. Immunohistochemical staining of brain tissue sections revealed a significant reduction in the number of Iba1^+^ microglia in KIN‐treated 5×FAD mice relative to vehicle‐treated controls, a finding indicative of attenuated microglial activation (Figure S6H,J; Figure S7A,B). To mechanistically validate this anti‐inflammatory activity, we assessed the expression of pro‐inflammatory mediators in Aβ‐stimulated primary microglia. Quantitative analysis demonstrated that KIN treatment significantly downregulated the mRNA expression of key pro‐inflammatory genes, including *Il‐6*, *Inos*, *Il‐1β* and *Tnf‐a*, in Aβ‐challenged primary microglia (Figure S7C–F). Collectively, these results demonstrate that KIN alleviates neuroinflammation in AD by dampening glial activation and suppressing pro‐inflammatory cytokine expression.

## 3 Discussion

Microglia‐driven neuroinflammation is increasingly recognized as an integral component of AD pathogenesis, and mounting evidence underscores its tight coupling to microglial metabolic dysfunction [9, 27]. As AD progresses, the brain's metabolic milieu undergoes profound remodeling, imposing severe bioenergetic constraints on microglia. In this context, nutrient availability and metabolic substrate partitioning have emerged as key determinants of microglial functional polarization—governing the switch between neuroprotective and neurotoxic phenotypes [6, 28]. Here, we demonstrate that elevated IDH1 expression in AD‐associated microglia leads to aberrant consumption of cytosolic citrate, depleting mitochondrial citrate input. This metabolic imbalance not only disrupts mitochondrial energy production but also H3K27 acetylation, thereby exacerbating microglial functional dysregulation. Through genetic ablation and pharmacological inhibition of IDH1, we show that suppressing IDH1 activity restores microglial metabolic homeostasis and mitigates neuroinflammatory responses. Notably, our small‐molecule screening identified KIN as a selective and potent IDH1 inhibitor that rescues AD‐related pathologies, positioning KIN as a promising therapeutic agent that acts by reprogramming microglial metabolism and suppressing neuroinflammation.

IDH1 is a cytosolic enzyme serving a central role in maintaining NADPH levels and supporting biosynthetic pathways by catalyzing the NADP^+^‐dependent oxidative decarboxylation of isocitrate to α‐KG [29, 30]. While IDH1 mutations have been extensively studied in cancer for their production of the oncometabolite 2‐HG and the resultant epigenetic dysregulation [31, 32], the role of WT IDH1 in neurodegeneration remains poorly defined. Our findings are the first to report the upregulation of WT IDH1 in AD microglia, where its enhanced activity induces compartmentalized metabolic disruptions. Specifically, we identify two pathological consequences of this dysregulation: mitochondrial citrate depletion, which impairs microglial bioenergetic capacity, and excessive cytosolic citrate consumption, which exacerbates H3K27ac‐mediated AD‐like pathologies. This dual disruption forms a feedforward loop between metabolic dysfunction and epigenetic reprogramming that sustains chronic microglial activation. Collectively, our results reveal WT IDH1 as a central node in the metabolic‐epigenetic regulatory network underlying AD pathogenesis. Targeting this node, in combination with key regulators of glycolysis and mitochondrial metabolism, may provide combinatorial therapeutic strategies to suppress chronic neuroinflammation and slow AD progression.

Recent studies from our group and others have implicated dysregulated TCA cycle activity and associated metabolite imbalances as key contributors to AD pathogenesis [12, 15]. Building on our previous work that established a glycolysis‐histone lactylation‐PKM2 regulatory loop in microglial activation [11], we now delineate a complementary mechanism wherein IDH1 overactivity perturbs mitochondrial TCA cycle dynamics. Unlike mutant IDH1 in tumors, elevated WT IDH1 in AD microglia depletes mitochondrial citrate—compromising TCA cycle flux and OXPHOS—while excessive cytosolic citrate consumption, which reinforces H3K27ac‐mediated AD‐like pathological damage. Thus, WT IDH1 emerges as a metabolic checkpoint linking mitochondrial dysfunction, epigenetic remodeling, and inflammatory activation in AD. Together with our previous finding, we conclude that upregulation of glycolysis (glycolysis–histone lactylation–PKM2 regulatory loop) and IDH1‐mediated citrate distribution disorder are of the two metabolic disorders during AD pathogenesis.

Despite recent advances in AD therapeutics, currently approved FDA treatments remain largely palliative, offering only symptomatic relief with minimal impact on disease progression [33, 34, 35, 36]. Given the established central role of IDH1 in AD pathogenesis, we sought to identify a selective IDH1 inhibitor capable of restoring microglial metabolic homeostasis and suppressing neuroinflammation. Through a multi‐tiered virtual screening campaign of 23,304 naturally occurring small molecules, we identified KIN as a potent and selective inhibitor of WT IDH1. Notably, unlike previously reported IDH1 inhibitors—which predominantly target mutant isoforms in cancer, structural modeling revealed that KIN occupies the isocitrate–metal ion‐binding site of WT IDH1, This binding mode enables KIN to form stabilizing hydrogen bonds with key catalytic residues, thereby competitively blocking IDH1 enzymatic activity. Functional validation assays further corroborated KIN's therapeutic potential: KIN treatment reactivated mitochondrial TCA cycle flux, improved microglial bioenergetic function, and normalized histone H3K27ac levels—core perturbations linked to AD‐associated microglial dysfunction. Collectively, these findings establish KIN as a promising disease‐modifying candidate for AD and provide a critical structural framework for the rational design of next‐generation WT IDH1‐targeted compounds.

Beyond its demonstrated efficacy in AD models, KIN has exhibited notable therapeutic potential in non‐neuronal disease contexts [37]. Specifically, KIN mitigates liver fibrosis and restores metabolic homeostasis in hepatic disease models, implying its biological effects may extend to other IDH1‐expressing tissues [38]. Whether KIN exerts these effects through mechanisms analogous to those identified in the CNS remains an open question that merits further investigation. Notably, as a natural product, KIN possesses inherent advantages over synthetic pharmaceutical agents—including structural diversity, reduced toxicity profiles, and favorable BBB permeability. These properties, coupled with its validated activity in AD models, underscore the translational potential of KIN or its derivatives for therapeutic development in AD and other IDH1‐associated disease contexts.

In conclusion, our study identifies IDH1 as a pivotal metabolic regulator in microglia, whose dysregulation directly contributes to AD pathogenesis. Notably, through a systematic small‐molecule screening campaign, we identified KIN, a natural compound, as a selective and potent inhibitor of WT IDH1. Both genetic ablation of Idh1 and pharmacological inhibition with KIN effectively restore microglial metabolic homeostasis, suppress pro‐inflammatory signaling cascades, and ameliorate core AD‐related pathologies, including Aβ deposition and cognitive deficits. Collectively, these findings highlight IDH1 as a validated and viable therapeutic target for AD and provide compelling preclinical evidence to support the development of metabolism‐based interventions for neurodegenerative diseases, with a particular focus on the natural product KIN.

## 4 Limitations of Study

While our findings support the therapeutic potential of IDH1 targeting and KIN, several limitations must be addressed. First, the pharmacokinetic properties of KIN—including its metabolic stability, bioavailability, and *in* *vivo* half‐life—were not fully characterized, and these activities are essential for advancing toward preclinical and clinical applications. Second, although we focused on microglial IDH1, KIN may also influence other IDH1‐expressing cell types, such as neurons and astrocytes. Evaluating these potential off‐target or synergistic effects will be critical for understanding the broader implications of pharmacological IDH1 inhibition. Third, while we observed KIN‐mediated modulation of citrate and H3K27ac levels, its broader epigenetic footprint—particularly regarding other histone marks and DNA methylation—remains unexplored. Finally, although we demonstrate IDH1 upregulation in AD brain tissue, longitudinal clinical studies are required to determine whether IDH1 dysfunction plays a causal role in disease onset and progression.

## 5 Experimental Section

### 5.1 Human Brain Samples

Frozen postmortem brain tissues were procured from the National Human Brain Bank for Development and Function, Chinese Academy of Medical Sciences & Peking Union Medical College (Beijing, China). The sample cohort included tissues from individuals diagnosed with AD and age‐matched neurologically healthy controls, with detailed demographic and clinical characteristics summarized in Table S4. All experimental procedures involving human tissues were approved by the Ethics Committee of the Beijing Institute of Basic Medical Sciences (Approval No. AF/SC‐08/02.320).

### 5.2 Mouse Models

Transgenic 5×FAD mice expressing human APP (695) harboring the Swedish (K670N/M671L), Florida (I716V), and London (V717I) mutations under the *Thy1* promoter, along with mutant human *PS1* (M146L and L286V), were used as an AD model, as previously described [11]. *Idh1* ^flox/flox^ (*Idh1* ^f/f^) mice were obtained from Cyagen Biosciences Inc., and *CX_3_CR_1_* ^CreERT2^ mice (Stock No: 021160) were purchased from The Jackson Laboratory (Sacramento, CA, USA). All transgenic lines were maintained in a C57BL/6J background. To generate conditional knockouts, *Idh1* ^f/f^ mice were crossed with CX_3_CR_1_^CreERT2^ and 5×.

*Idh1* ^f/f^, *Idh1* ^f/f^; *CX_3_CR_1_* ^CreERT2^, *Idh1* ^f/f^; 5×FAD, and *Idh1* ^f/f^; *CX_3_CR_1_* ^CreERT2^; 5×FAD. Genotyping was performed using PCR on DNA extracted from tail biopsies.

For pharmacological intervention studies, the first cohort of male‐only 5×FAD mice was randomly assigned to either the KIN treatment group or the vehicle control group at 2.5 months of age. KIN was dissolved in 0.9% sterile saline and administered intragastrically at a dose of 50 mg/kg/day for 2.5 consecutive months, until the end of behavioral testing at 5 months of age. Vehicle control mice received an equal volume of 0.9% sterile saline under the same schedule. The second cohort of male‐only 5×FAD mice was used in the experiments. At 25 weeks of age, mice were randomly assigned to either the KIN treatment group or the vehicle control group to initiate pharmacological intervention. KIN was dissolved in 0.9% sterile saline and administered intragastrically at a consistent dose of 50 mg/kg/day, with dosing maintained from 25 weeks until 30 weeks of age.

Conditional deletion of *Idh1* in microglia was induced by tamoxifen in male and female mice, with equal sex ratios in each experimental group. Tamoxifen (S1238, Selleck) was dissolved in corn oil (C8267, Sigma‐Aldrich) at 20 mg/mL. At 2 months of age, mice received intragastric administration of 200 µL tamoxifen daily for five consecutive days. Behavioral analyses were performed with 8 male mice and 7 female mice per group, while pathological analyses were conducted on mice with equal sex ratios in each group, all at 3 months pos‐induction (in 5‐month‐old mice). All mice were housed in groups of 4–5 per cage under standard conditions (12‐h light/dark cycle, 22°C) with ad libitum access to autoclaved water and standard rodent chow (SPF‐F01‐002, SPF Beijing Biotechnology). Experimental cohorts consisted of age‐ and sex‐matched C57BL/6J background mice. Sentinel health monitoring was conducted every three months, and all mice remained healthy throughout the study. All animal procedures were approved by the Institutional Animal Care and Use Committee of the Beijing Institute of Basic Medical Sciences.

### 5.3 Primary Microglia Cultures

Primary microglia were isolated from neonatal C57BL/6J mice (postnatal days 0–3). Mouse brains were rapidly harvested, and meninges were carefully removed in ice‐cold 1× phosphate‐buffered saline (PBS). Brain tissues were enzymatically dissociated with 0.25% trypsin (Biological Industries) at 37°C for 15–20 min, followed by gentle trituration in Dulbecco's modified Eagle's medium (DMEM) supplemented with 10% fetal bovine serum (FBS; Biological Industries) and 1% penicillin‐streptomycin (Sigma‐Aldrich). Dissociated cells were plated onto poly‐L‐ornithine‐coated cell culture flasks. After 3 days in culture, the medium was replaced with growth medium consisting of DMEM supplemented with 20% FBS, 1 ng/mL mouse fibroblast growth factor (FGF; cat. no. 450‐33, Peprotech), and 1 ng/mL mouse β‐nerve growth factor (β‐NGF; cat. no. 450‐34, Peprotech). Following 10–14 days of culture, microglia were isolated from mixed glial cultures via gentle mechanical agitation and immediately used for subsequent experimental procedures.

### 5.4 Adult Microglia Isolation

Microglia were isolated from the brains of adult mice at the indicated ages. Mice were deeply anesthetized with sodium pentobarbital, followed by transcardial perfusion with ice‐cold physiological saline. Brains (cerebellum excluded) were dissected and rinsed in ice‐cold phosphate‐buffered saline (PBS). Tissue dissociation was performed using the Adult Brain Dissociation Kit (cat. no. 130‐107‐677, Miltenyi Biotec) strictly according to the manufacturer's instructions. Myelin debris was removed using the Cell Debris Removal Buffer provided in the kit, and the resulting cell suspensions were resuspended in PBS supplemented with 0.5% bovine serum albumin (BSA). Microglia were enriched via magnetic‐activated cell sorting (MACS) using anti‐CD11b MicroBeads (cat. no. 130‐093‐634, Miltenyi Biotec) and MACS Multistand separators, following the manufacturer's standardized protocol. Freshly isolated microglia were used immediately for LC‐MS analyses. For RNA or protein extraction, isolated cells were flash‐frozen in liquid nitrogen and stored at –80°C until use.

### 5.5 Morris Water Maze

Spatial learning and memory were evaluated using the Morris water maze paradigm. Mice were habituated to the test apparatus (a circular pool with a diameter of 110 cm) 24 h prior to the initiation of training. The pool was filled with opaque water maintained at a constant temperature of 19°C–22°C and arbitrarily divided into four equal quadrants. A hidden escape platform (10 cm in diameter) was submerged 1 cm below the water surface and positioned in a fixed quadrant throughout the training phase. Distinct visual cues (varying in shape and color) were affixed to the walls of each quadrant to provide spatial reference information for navigation. During daily training, each mouse was given a maximum of 60 s to locate the hidden platform. Mice that failed to find the platform within the allotted time were gently guided to it and allowed to remain on the platform for 30 s to consolidate spatial memory. Each mouse completed two training trials per day, and performance metrics were averaged across trials. Memory retention was assessed in a probe trial conducted 24 h after the final training session, during which the hidden platform was removed. Mouse swimming trajectories were recorded in real time via video tracking and analyzed using ANY‐maze software (Stoelting, Wood Dale, IL, USA) to quantify spatial memory‐related behaviors.

### 5.6 Novel Object Recognition

Recognition memory was evaluated via the novel object recognition (NOR) assay, which consists of habituation, familiarization, and test phases. Mice were first habituated to the behavioral testing environment over three consecutive days: each mouse was individually placed in a rectangular arena (50 × 50 × 20 cm, object‐free) and allowed to explore freely for 5 min per day to minimize neophobia. On the fourth day (familiarization phase), two identical objects were placed symmetrically in the arena, and each mouse was allowed to explore the objects for 5 min. Following a 1‐h retention interval, the test phase was conducted: one of the familiar objects was replaced with a novel object of distinct shape and texture, and mice were again placed in the arena for a 5‐min exploration period. All behavioral sessions were recorded using an overhead video camera, and exploration behaviors were analyzed using ANY‐maze tracking software (Stoelting, Wood Dale, IL, USA). A recognition index was calculated to quantify recognition memory, defined as the time spent exploring the novel object divided by the total time spent exploring both the novel and familiar objects.

### 5.7 ATP Measurement

Intracellular ATP levels were quantified using a previously established protocol [11]. Briefly, the culture medium was carefully aspirated from primary microglial cultures. Cells were then lysed in 100 µL of lysis buffer containing luciferase reagents (as provided in the assay kit). Following a 10‐min incubation at room temperature to ensure complete lysis and reagent reaction, luminescence intensity was measured using a microplate reader. ATP concentrations were calculated based on a standard curve and normalized to the total protein content of each sample to account for differences in cell number.

### 5.8 DARTS Assay

Drug Affinity Responsive Target Stability (DARTS) assays were performed as follows: cell lysates (containing ∼100 µg total protein) were incubated with either kinsenoside (KIN) or vehicle (dimethyl sulfoxide, DMSO) for 25 min at 25°C. Pronase (cat. no. 53702, Sigma–Aldrich) was then added to the reaction system, and samples were subjected to proteolytic digestion for 15 min at room temperature. Proteolysis was terminated by adding a protease inhibitor cocktail, followed by a 10‐min incubation on ice to ensure complete inhibition. SDS‐PAGE loading buffer was subsequently added to each sample, which were then denatured at 70°C for 10 min prior to sodium dodecyl sulfate‐polyacrylamide gel electrophoresis (SDS‐PAGE).

### 5.9 Cloning, Expression, and Purification of IDH1

The pET28a vector harboring the IDH1 gene (pET28a‐IDH1) was a kind gift from Dr. Qi Xie (Westlake University). To generate the expression construct, the IDH1 coding sequence was amplified from human genomic DNA via polymerase chain reaction (PCR). The amplified fragment was engineered to contain an N‐terminal hexahistidine (His) tag and a tobacco etch virus (TEV) protease cleavage site before subcloning into the pET28a vector. The recombinant pET28a‐IDH1 plasmid was transformed into E. coli T7 expression cells (cat. no. BC206‐02, Biomed). Transformed bacteria were cultured in Luria‐Bertani (LB) medium supplemented with 50 µg/mL kanamycin at 37°C with shaking until the optical density at 600 nm (OD_600_) reached 0.4–0.6. The culture temperature was then shifted to 20°C, and recombinant protein expression was induced with 0.5 mm isopropyl β‐D‐1‐thiogalactopyranoside (IPTG; cat. no. I1020, Solarbio) for 16 h at 200 rpm. Bacterial cells were harvested and lysed by high‐pressure homogenization. The cell lysate was centrifuged at 12 000 × g for 30 min at 4°C to remove insoluble debris. The supernatant containing soluble His‐tagged IDH1 was loaded onto a Ni‐nitrilotriacetic acid (Ni‐NTA) affinity chromatography column. After extensive washing, the target protein was eluted with elution buffer containing 150 mM imidazole. The purity of the eluted IDH1 protein was verified by SDS‐PAGE followed by Coomassie brilliant blue staining.

### 5.10 Biolayer Interferometry

Binding interactions between IDH1 and KIN were evaluated via biolayer interferometry (BLI) using the Octet RED96 system (Pall Life Sciences). Prior to the assay, Octet streptavidin (SSA) biosensors were pre‐equilibrated in assay buffer (PBS pH 7.9 supplemented with 0.02% Tween‐20) for 10 min to stabilize baseline signals. Biotinylated His‐tagged IDH1 protein was immobilized onto the pre‐equilibrated SSA biosensors by immersing the sensors in 96‐well plates containing 200 µL/well of 0.1 mg/mL biotinylated IDH1 in assay buffer. Following immobilization, unbound protein was thoroughly washed away with assay buffer, and residual streptavidin binding sites on the biosensors were blocked with biocytin to minimize nonspecific binding. KIN (cat. no. DJ0053‐0010, PUSH) was serially diluted in assay buffer to generate six concentrations ranging from 0.94 to 30 µm. For kinetic measurements, the IDH1‐immobilized biosensors were sequentially exposed to each KIN concentration and fresh assay buffer, with each phase monitored for 120 s. Throughout the experiment, the biosensors were agitated at 1000 rpm, and the system temperature was maintained at 30°C. Reference biosensors were processed in parallel to account for nonspecific interactions between KIN and the biosensor matrix. Between experimental runs, biosensors were regenerated using assay buffer (PBS pH 7.9 + 0.02% Tween‐20), and a new baseline was established to ensure data reliability. BLI data were corrected by subtracting reference sensor signals, and binding kinetics were analyzed using the Octet Data Analysis software. The equilibrium dissociation constant (K) was calculated based on the steady‐state response values, assuming a 1:1 ligand‐receptor interaction model.

### 5.11 Virtual Screening

A five‐step virtual screen identified potent IDH1 inhibitors from natural products, as follows: 1. Library Prep 23 304 natural compounds were retrieved from TCMD‐2009 (NeoTrident Technology LTD) in SDF format (annotated with name, ID, and structure). 2. BBB Permeability AD's CNS tropism prompted BBBper model evaluation (RDKit‐calculated descriptors: MW, HBDs/HBAs, xLogP, TPSA, etc.). Compounds with BBBper > 0.6 (favorable penetration) were retained [39]. 3. Drug‐Likeness Lipinski's RO5 compliance (MW < 500, LogP < 5, HBDs < 5, HBAs < 10, rotatable bonds < 10; RDKit) ensured favorable pharmacokinetics [40]. 4. Substrate Similarity Cosine similarity to isocitrate (IDH1 substrate) was calculated; candidates with similarity > 0.95 (substrate‐mimetic) were prioritized. 5. Docking & Hit Selection Surflex‐Dock (Sybyl‐X) docked compounds to IDH1 (PDB: 5YFN, preprocessed). Hits with Total_Score > 6 (strong binding) were ranked by logP (favoring solubility for IDH1's hydrophilic pocket) for validation.

### 5.12 Construction of Brain‐Specific IDH1 Co‐Expressed Gene Module

Transcriptional Data Acquisition and Processing Transcriptional data were retrieved from the National Center for Biotechnology Information (NCBI) Gene Expression Omnibus (GEO) database, with a focus exclusively on microarray datasets. Human AD transcriptional data were obtained from the GEO dataset GSE36980, which comprises 80 postmortem human brain tissue samples spanning three brain regions: hippocampus, temporal cortex, and frontal cortex. Raw data (CEL files) were uniformly processed using platform‐specific pipelines for filtering and normalization. For each brain region, differential gene expression profiles between AD and non‐AD samples were generated using the R package “Limma” (v3.32.7). Each gene expression profile was then used to construct an ordered gene list, sorted in descending order based on differential expression values (AD vs. non‐AD). Construction of Brain‐Specific IDH1 Co‐Expressed Gene Module (BIM). The brain‐specific IDH1 co‐expressed gene module (BIM) was established by integrating IDH1 with brain‐specific IDH1 co‐expressed genes (detailed in Table S1). Co‐expressed genes were identified using the Search‐Based Exploration of Expression Compendium (SEEK; http://seek.princeton.edu/) platform, which employs a query‐level cross‐validation algorithm [41]. Only brain tissue‐derived co‐expressed genes with a *p* value < 0.01 were included in the final BIM.

### 5.13 Enrichment Analysis of BIM in Transcriptional Profiles

The characterization of BIM in AD‐induced transcriptional profiles was evaluated using gene set enrichment analysis (GSEA), and implemented in the R package, “GSEA‐P” [42]. GSEA took BIM and the order gene lists of AD as inputs to calculate enrichment scores (ES) of BIM in gene expression profiles using the Kolmogorov–Smirnov statistics. The ES values with in [‐1 1] could evaluate the overrepresentation of BIM at the extremes (bottom or top) of each transcriptional profile.

### 5.14 Immunohistochemistry

Mouse brains were sectioned coronally at 40 µm thickness using a cryostat (Leica CM3050S) and preserved at −20°C in cryoprotective solution (300 g sucrose, 10 g polyvinylpyrrolidone, 500 mL 0.1m phosphate buffer, 300 mL ethylene glycol, and ddH_2_O to 1 L) until further use. Before staining, sections were washed three times in PBS and blocked at room temperature for 1.5 h with buffer containing 0.3% Triton X‐100, 5% horse serum, and 2% BSA in PBS. Primary antibody incubation was performed overnight at 4°C. The following primary antibodies were used: rabbit monoclonal anti‐IDH1 (A24133, ABclonal), mouse monoclonal anti‐6E10 (803001, Biolegend), goat polyclonal anti‐Iba1 (NB100‐1028, Novus), chicken polyclonal anti‐GFAP (AP31806PU‐N, Origene), and anti‐Acetyl‐Histone H3 (Lys27) Rabbit mAb (PTM‐116RM, PTM BIO). For immunofluorescence co‐labeling, fluorescent secondary antibodies were applied as follows: Alexa Fluor 568 donkey anti‐Rabbit IgG (A10042), Alexa Fluor 647 donkey anti‐Chicken IgY (A78952), and Alexa Fluor 488 donkey anti‐Goat IgG (A11055), all from Thermo Fisher. Fluorescent images were captured using a Nikon confocal microscope.

For IDH1 staining, sections underwent heat‐induced epitope retrieval in 50 mm sodium citrate with 0.05% Tween‐20 at 95°C for 30 min, followed by triple PBS washes prior to immunofluorescence staining.

### 5.15 Thioflavin S (TS) Staining

To visualize Aβ plaques, TS staining was performed by incubating brain sections in 0.002% TS (T1892‐25G, Sigma‐Aldrich) prepared in 50% ethanol for 8 min in the dark. Sections were then washed twice with 50% ethanol and three times with PBS before mounting for imaging or undergoing additional immunofluorescence staining.

### 5.16 Preparation of Oligomeric Aβ_1‐42_

Human Aβ_1‐42_ peptides (cat. no. AS‐20276, AnaSpec) were initially solubilized in 1,1,1,3,3,3‐hexafluoro‐2‐propanol (HFIP) to disrupt preformed aggregates. The peptide solution was then vacuum‐lyophilized at 4°C to form a homogeneous peptide film. This film was reconstituted in dimethyl sulfoxide (DMSO) to a final concentration of 1 mm, and the stock solution was stored at −80°C until use. To generate oligomeric Aβ_1‐42_, the 1 mm DMSO stock was diluted to 10 µm in Dulbecco's modified Eagle's medium (DMEM). The diluted peptide solution was incubated at 37°C overnight to induce oligomer formation.

### 5.17 Western Blotting

Protein lysates were prepared from brain tissues or cultured cells using RIPA lysis buffer supplemented with protease and phosphatase inhibitor cocktails. Protein concentrations were determined via BCA assay, and equal protein loading was ensured by normalizing concentrations prior to SDS‐PAGE. Equal amounts of protein were separated by SDS‐PAGE and electrophoretically transferred to polyvinylidene difluoride (PVDF) membranes (Millipore). Membranes were blocked with 5% non‐fat milk in Tris‐buffered saline with 0.1% Tween‐20 (TBST) for 1 h at room temperature to minimize non‐specific binding. Subsequently, membranes were incubated with primary antibodies overnight at 4°C, followed by incubation with horseradish peroxidase (HRP)‐conjugated secondary antibodies at room temperature. Protein signals were detected using an enhanced chemiluminescence (ECL) detection system and visualized on Kodak autoradiography film. The primary antibodies used in this study were as follows: anti‐IDH1 (cat. no. A24133, ABclonal), anti‐IDH2 (cat. no. A5106, ABclonal), anti‐6E10 (cat. no. 803001, BioLegend), and anti‐β‐actin (cat. no. 66009‐1‐Ig, Proteintech).

### 5.18 RT‐qPCR

Total RNA was extracted from tissues or cultured cells using Trizol reagent (15596‐026, Thermo Fisher), and cDNA was synthesized using a one‐step First‐Strand cDNA Synthesis Kit (Transgen). Gene expression was assessed by SYBR Green‐based qPCR. Primer sequences are listed in Table S6. Expression levels were normalized to β‐actin.

### 5.19 Seahorse Extracellular Flux Assays

The ECAR and OCR of primary microglia were quantified using a Seahorse XFe 96 Extracellular Flux Analyzer (Seahorse Bioscience). ECAR and OCR were measured with the Seahorse XF Glycolysis Stress Test Kit (cat. no. 103020‐100; Agilent Technologies) and Seahorse XF Cell Mito Stress Test Kit (cat. no. 103015‐100; Agilent Technologies), respectively, strictly following the manufacturer's protocols. Briefly, primary microglia were seeded into a Seahorse XF 96‐well culture plate at a density of 2 × 10^4^ cells per well and cultured overnight. Cells were then treated with the indicated drug for 24 h before metabolic measurements. Prior to the assay, culture medium was aspirated, and cells were washed twice with pre‐warmed XF assay medium, followed by incubation in the same medium to equilibrate. For ECAR analysis, baseline measurements were acquired first, then glucose (1 mm), oligomycin (1 µm), and 2‐deoxyglucose (2‐DG, 50 mm) were sequentially injected into the wells at specified time points. For OCR analysis, after baseline recording, oligomycin (1 µm), carbonyl cyanide‐p‐trifluoromethoxyphenylhydrazone (FCCP, 2 µm), and a mixture of rotenone and antimycin A (Rote/AA, 0.5 µm each) were injected in sequence. Metabolic data were collected and analyzed using Seahorse XF 96 Wave software. ECAR values were expressed as mpH/min, and OCR values as pmol/min, with both normalized to the total protein concentration of each well to account for differences in cell number.

### 5.20 Mitochondrial Isolation

Mitochondria were isolated from microglia using a modified protocol based on the Mitochondria Isolation Kit (cat. no. 89874, Thermo Fisher Scientific). Fresh brain tissues were minced in ice‐cold homogenization buffer, enzymatically digested with 0.25% trypsin at 37°C for 15 min, and gently triturated through a 70 µm cell strainer to generate a single‐cell suspension. Microglia were enriched from the single‐cell suspension via magnetic‐activated cell sorting (MACS) using anti‐CD11b MicroBeads (cat. no. 130‐093‐634, Miltenyi Biotec). Purified microglia (1 × 10^7^ cells) were resuspended in chilled isolation buffer and homogenized on ice with 30 strokes using a Dounce homogenizer. The homogenate was mixed with Buffer C and centrifuged at 800 × g for 10 min at 4°C to pellet nuclei and cellular debris. The resulting supernatant was collected and centrifuged at 12 000 × g for 15 min at 4°C to sediment mitochondria. The mitochondrial pellet was washed twice with isolation buffer and resuspended in mitochondrial assay buffer. The remaining supernatant after mitochondrial pelleting was collected as the cytosolic fraction.

### 5.21 IDH Activity Assay

IDH enzymatic activity was quantified using the IDH Assay Kit (cat. no. MAK062, Sigma–Aldrich) in strict accordance with the manufacturer's instructions. Three IDH isoforms were targeted, distinguished by their subcellular localization and cofactor specificity: cytosolic IDH1 (NADP^+^‐dependent), mitochondrial IDH2 (NADP^+^‐dependent), and mitochondrial IDH3 (NAD^+^‐dependent).

To specifically assess IDH2 activity, NADP^+^ was used as the cofactor in reactions containing mitochondrial extracts. For sample preparation, microglia were harvested and subjected to subcellular fractionation to isolate cytosolic and mitochondrial components. Isolated mitochondria were lysed in IDH assay buffer, and IDH enzymatic activity was measured using isocitrate as the substrate—this reaction generates a colorimetric product (absorbance peak at 450 nm) whose intensity is proportional to enzyme activity. Reactions were incubated at 37°C for 3 min to initiate the enzymatic reaction, followed by kinetic monitoring of optical density at 450 nm at 5‐min intervals. IDH activity was calculated as the amount of NADH (for IDH3) or NADPH (for IDH1/2) produced per minute per million microglia, normalized to cell number for quantitative comparison.

### 5.22 Citrate and α‐KG Quantification

Cytosolic supernatants were collected via centrifugation at 13 000 × g for 10 min. Concentrations α‐KG and citrate in these supernatants were quantified using the α‐KG Assay Kit (cat. no. MAK054, Sigma–Aldrich) and Citrate Assay Kit (cat. no. BC2155, Solarbio), respectively. For mitochondrial metabolite analysis, mitochondrial pellets were resuspended in α‐KG assay buffer and homogenized using a Dounce homogenizer. The homogenate was centrifuged at 13 000 × g for 10 min, and the resulting supernatant was used for subsequent measurements. For both assays, 50 µL of sample (cytosolic or mitochondrial supernatant) was transferred to a black‐walled, clear‐bottom 96‐well plate, followed by the addition of 50 µL of kit‐specific reaction mixture. Plates were gently mixed on a horizontal shaker and incubated at 37°C for 30 min under light‐protected conditions to prevent signal degradation. Concentrations of α‐KG and citrate were calculated by interpolating sample signals against their respective standard curves generated according to the kit protocols.

### 5.23 Transcriptomic Analysis

mRNA Library Construction and Sequencing​. Total RNA was isolated using TRIzol reagent (Invitrogen) and quantified via NanoDrop ND‐1000. RNA integrity was verified by Agilent 2100 Bioanalyzer (RIN > 7.0) and denaturing agarose gel electrophoresis. Poly(A) RNA (1 µg total RNA input) was purified with Dynabeads Oligo (dT) 25 (Thermo Fisher) via two rounds of enrichment, then fragmented using Magnesium RNA Fragmentation Module (NEB) at 94°C for 5–7 min.​ Cleaved RNA was reverse‐transcribed to cDNA with SuperScript II Reverse Transcriptase (Invitrogen), followed by synthesis of U‐labeled second‐stranded DNAs using E. coli DNA polymerase I, RNase H (both NEB), and dUTP Solution (Thermo Fisher). Blunt ends were A‐tailed for indexed adapter ligation; size selection was performed with AMPure XP beads. After U‐labeled second‐strand digestion by heat‐labile UDG (NEB), ligated products were amplified via PCR: 95°C for 3 min (initial denaturation), 8 cycles of 98°C for 15 sec, 60°C for 15 sec, 72°C for 30 sec, and final extension at 72°C for 5 min. The final cDNA library had an average insert size of 300 ± 50 bp. Sequencing was conducted as 2×150 bp paired‐end reads (PE150) on an Illumina NovaSeq 6000 (LC‐Bio Technology).

Adaptor‐contaminated reads were removed using Cutadapt v1.9 (‐a ADAPT1 ‐A ADAPT2 ‐O 5 ‐m 100). After filtering low‐quality/undetermined bases, clean reads were mapped to the Homo sapiens Ensembl v96 genome via HISAT2 v2.0.4. Mapped reads were assembled with StringTie v1.3.4d; all sample transcriptomes were merged using gffcompare v0.9.8 to generate a comprehensive transcriptome.​ mRNA expression levels (FPKM) were quantified by StringTie and Ballgown. Differentially expressed mRNAs were identified using edgeR or DESeq2 with thresholds: fold change > 2 or < 0.5, and p < 0.05.

### 5.24 Metabolite Extraction and LC‐MS Analysis

Metabolic standards were sourced from Sigma–Aldrich (St. Louis, MO, USA), Steraloids Inc. (Newport, RI, USA), and TRC Chemicals (Toronto, ON, Canada). Standards were accurately weighed and dissolved in water, methanol, NaOH, or HCl to prepare 5.0 mg/mL stock solutions, which were combined to generate calibration mixtures. All solvents—including formic acid (Optima grade), methanol, acetonitrile, and isopropanol (Optima LC‐MS grade)—were obtained from Thermo Fisher Scientific (Fair Lawn, NJ, USA). Ultrapure water was produced using a Milli‐Q Reference system with an LC‐MS Pak filter (Millipore, Billerica, MA, USA). Each cell pellet, stored in an Eppendorf Safelock microcentrifuge tube, was mixed with 10 pre‐chilled zirconium oxide beads and 20 µL of deionized water, then homogenized for 3 min. Subsequently, 150 µL of methanol containing an internal standard was added for metabolite extraction, followed by another 3 min of homogenization and centrifugation at 18 000 × *g* for 20 min. The resulting supernatant was transferred to a 96‐well plate.

Downstream steps were carried out using an Eppendorf epMotion Workstation (Eppendorf Inc., Hamburg, Germany). A freshly prepared derivatization reagent (20 µL) was added to each well, and the plate was sealed and incubated at 30°C for 60 min. After derivatization, samples were evaporated for 2 h. Ice‐cold 50% methanol (330 µL) was then added for reconstitution. Plates were stored at −20°C for 20 min and centrifuged at 4°C, 4000 × *g* for 30 min. A 135 µL aliquot of supernatant was transferred to a new 96‐well plate containing 10 µL internal standard per well. Serial dilutions of derivatized standard solutions were added to designated wells. The plate was sealed and prepared for LC‐MS analysis.

Differential metabolites were subjected to KEGG pathway enrichment analysis. First, metabolites were annotated to obtain KEGG IDs. Then, a hypergeometric test was employed to calculate the probability of differential metabolites enriched in each pathway, with the total detected metabolites as the reference set. Multiple testing correction, such as the Benjamini—Hochberg method, was applied to control the false discovery rate. Pathways with adjusted *p* values < 0.05 were considered significantly enriched, and were visualized using tools like KEGG mapper for further biological interpretation.

### 5.25 Crystallization, Data Collection, and Structure Determination of the IDH‐KIN Complex

Recombinant IDH1 protein was incubated with compound KIN at a molar ratio of 1:10 for 2 h at 4°C prior to crystallization. Screening was performed using both commercial and in‐house sparse‐matrix kits via the hanging‐drop vapor diffusion method at 16°C. Optimal crystals formed in a solution containing 0.5 m ammonium sulfate, 0.1 m MES (pH 6.0), and 15% (w/v) PEG4000, with protein concentration at 20 mg/mL. Crystals were cryoprotected in 20% (v/v) ethylene glycol and flash‐frozen in liquid nitrogen.

X‐ray diffraction data were collected at beamline BL‐10U2 of the Shanghai Synchrotron Radiation Facility (SSRF) using a CCD detector. Data processing was performed using HKL3000 [43]. The structure was solved by molecular replacement with PHENIX [44], using PDB entry 3MAP as the template. Structural figures were generated in PyMOL (http://pymol.org). Data collection and refinement statistics are summarized in Table S3.

### 5.26 Accession Codes

Coordinates and structure factors for the IDH1‐KIN complex have been deposited in the Protein Data Bank under accession code 9M82.

### 5.27 Statistical Analysis

All quantitative data are presented as the mean ± standard error of the mean (SEM). Statistical analyses were performed using GraphPad Prism software (version 9.0). For group comparisons, unpaired Student's *t*‐tests were used for two‐group analyses, while one‐way or two‐way analysis of variance (ANOVA) followed by Tukey's post hoc test was applied for multiple‐group comparisons, as appropriate. Statistical significance was defined as a *p* value < 0.05.

## Author Contributions

Z.Y., Y.L., Q.H., and R.‐Y.P. conceived and supervised the project. Q.L. designed and performed the majority of experiments. Q.L., Y.L., R.‐Y.P., and Z.Y. analyzed data, and wrote the manuscript with input from all authors. Y.L. directed the recombinant IDH1 protein expression and purification. Y.L. and S.L. assisted in IDH1 protein expression and purification. S.O.Y., Z.L., Y.X., and Y.‐B.Z. conducted co‐crystallization of IDH1‐KIN complexes and structural determination. H.W. and T.J. carried out animal genotyping and behavioral analyses. P.L. executed the virtual screening of IDH1 inhibitors. All authors critically reviewed and approved the final manuscript.

## Conflicts of Interest

Z.Y., R.‐Y.P., and Y.L. are inventors on three Chinese patents related to this work (Patent Nos. ZL201911115335.7, ZL201911114214.0, and ZL201911114215.5). Other authors declare no competing interests.

## Supporting information




**Supporting File 1**: advs75125‐sup‐0001‐SuppMat.docx.

**Supporting File 2**: advs75125‐sup‐0002‐Table S1 and S2.docx.

**Supporting File 3**: advs75125‐sup‐0003‐Uncropped blots.zip.

## Data Availability

Uncropped Western blotting images and raw data used for figure generation are provided in Supplementary Information. Additional details necessary to replicate or reanalyze the data reported in this paper are available upon request from the lead author.
