# Supplementary material for: Kinsenoside Targets IDH1 to Restore Microglial Immune‐Metabolic Homeostasis for Alzheimer's Disease Therapy
Source: Adv Sci (Weinh). 2026 Apr 14;13(36):e75125. doi: 10.1002/advs.75125 (PMC13317708; doi:10.1002/advs.75125)
Supplement: Supplementary file 1 — Supporting File 1: advs75125‐sup‐0001‐SuppMat.docx. [file ADVS-13-e75125-s002.docx]

**Figure S1. *IDH1* mRNA expression in brain tissue from patients with AD**

(A–D) Analysis of *IDH1* mRNA levels in clinical samples from patients with AD (*n* = 22) and healthy controls (*n* = 8) using the GEO dataset GSE28146.

(E–G) qPCR quantification of *IDH1* mRNA in postmortem brain sections from patients with AD and healthy controls (*n* = 5 per group).

Data are presented as mean ± SEM. **p* < 0.05, ***p* < 0.01. Statistical comparisons were made using two-tailed unpaired Student’s *t*-test (E–G) or one-way ANOVA (A–D) with Tukey’s multiple comparisons test.


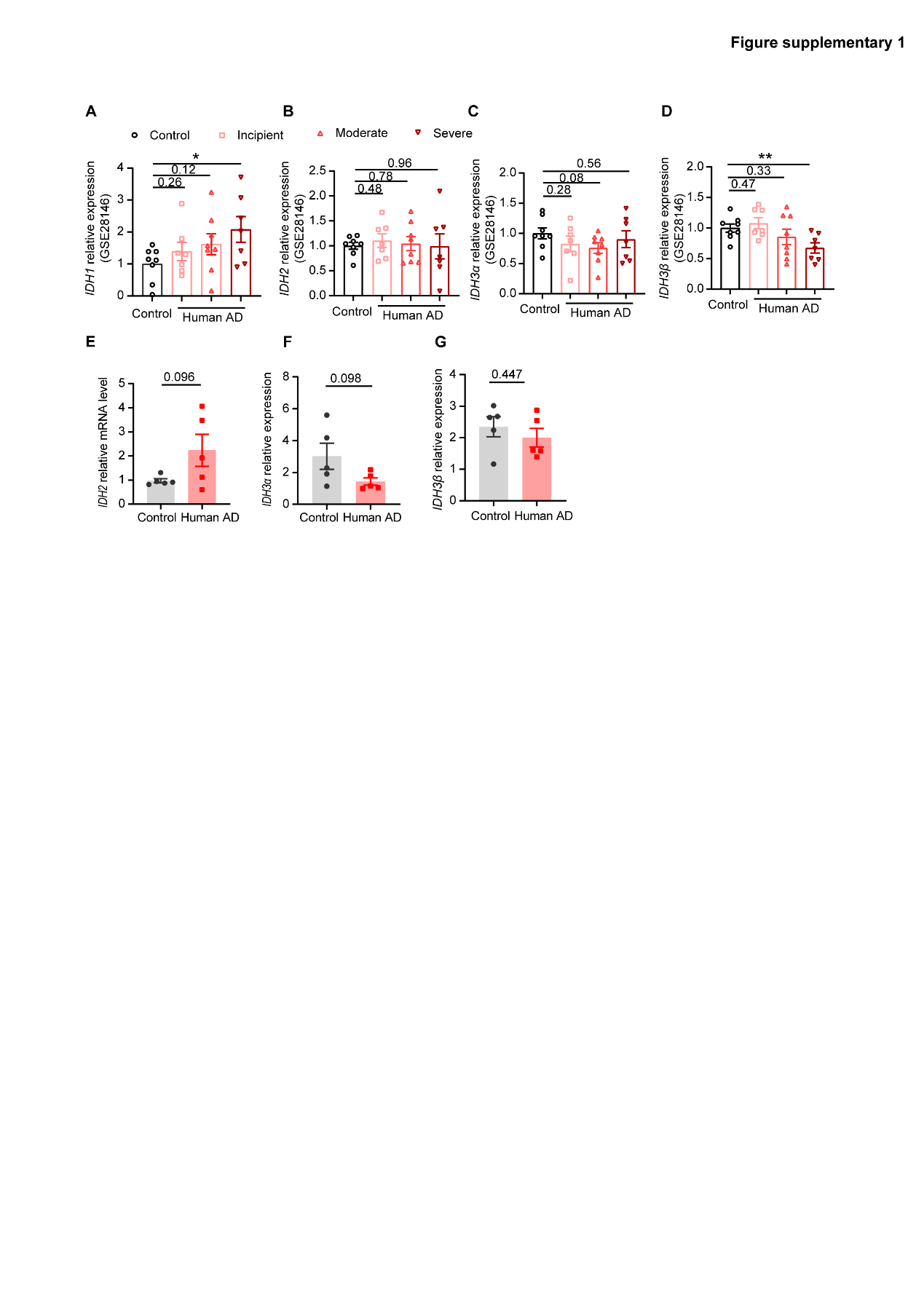


**Figure S2. Ablation of IDH1 in microglia suppresses neuroinflammation**

(A) Relative *Idh1* mRNA expression in primary cultured microglia transfected with siNC or siIDH1, measured by qPCR.

(B–D) qPCR analysis of *Il-6*, *Il-1β*, and *Tnf-α* expression in microglia transfected with siNC or siIDH1 and stimulated with Aβ for 3 or 6 hours (*n* = 3 per group).

Data are presented as mean ± SEM. *****p* < 0.0001; N.S., not significant. Statistical comparisons were made using two-tailed unpaired Student’s *t*-test (A–D).


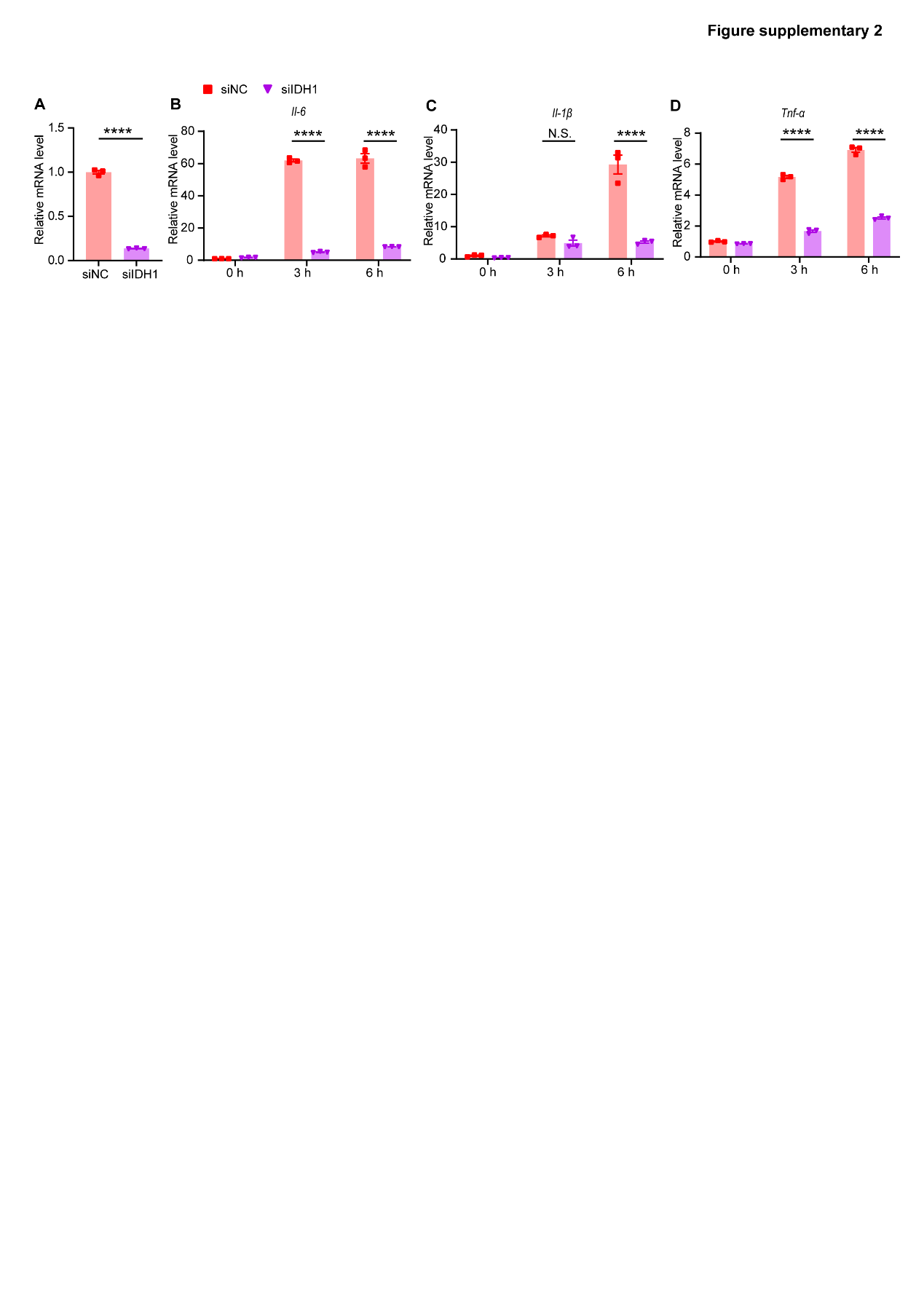


**Figure S3. Ablation of IDH1 in microglia increased histone H3K27ac levels**

(A) Representative immunofluorescence images showing H3K27ac co-stained with IBA1 in the CA1 region of 5-month-old f/f;AD and cKO;AD mice.

(B) Quantification of H3K27ac intensity in Iba1⁺ microglia from panel (A) (*n* = 4 per group).

Data are presented as mean ± SEM. **p* < 0.05, ***p* < 0.01. Statistical comparisons were made using one-way ANOVA (B) with Tukey’s multiple comparisons test.


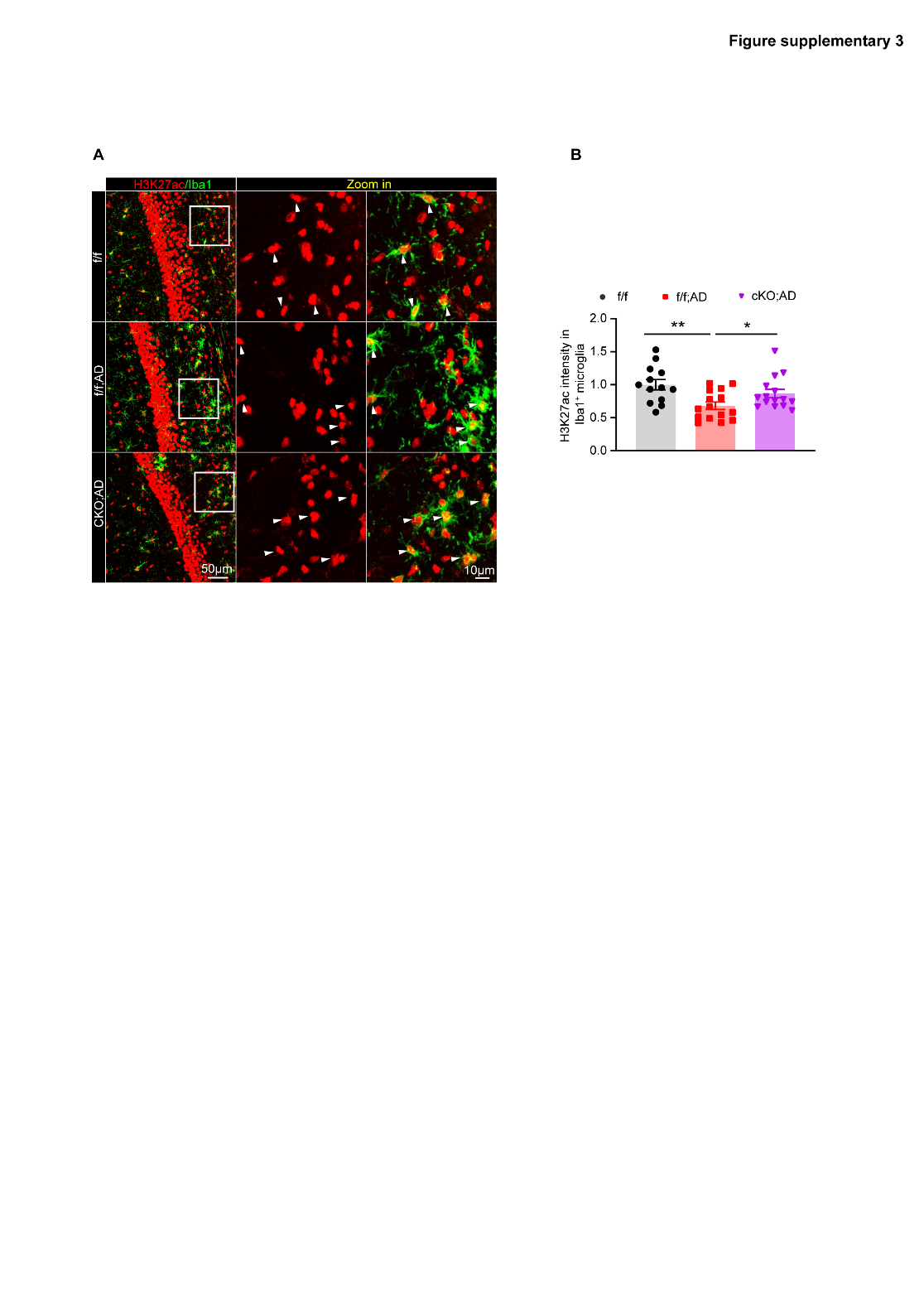


**Figure S4. Identification and validation of KIN as a selective IDH1 inhibitor**

(A) Candidate compounds were screened for BBB permeability (threshold: BBB > 0.6).

(B) Compounds were evaluated for drug-likeness using Lipinski’s Rule of Five.

(C) Structural similarity to isocitrate was assessed by cosine similarity (threshold > 0.95).

(D) Molecular docking scores were used to rank compounds by binding affinity to IDH1 (threshold > 6).

(E) Compounds were also assessed for aqueous solubility.


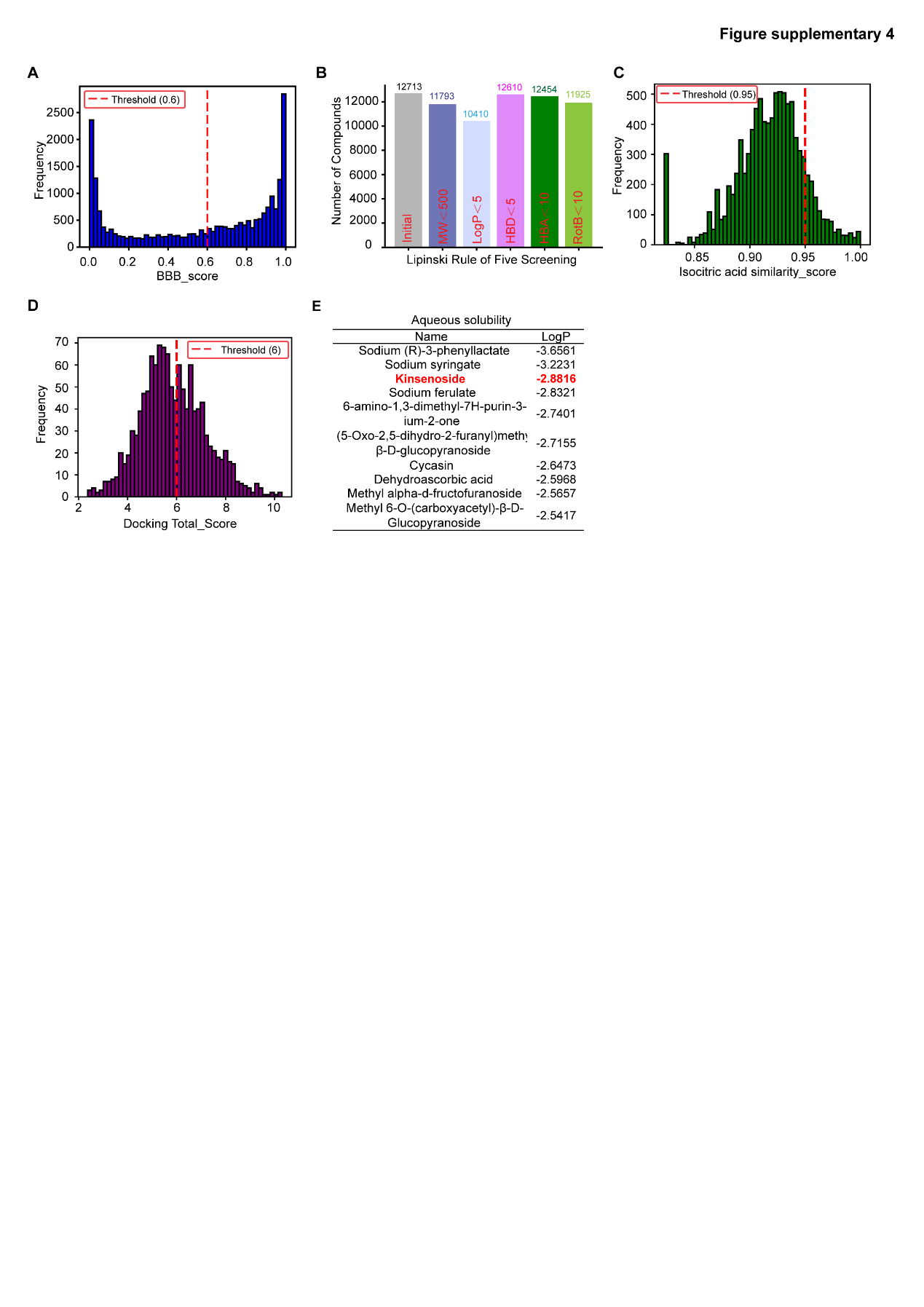


**Figure S5. KIN increased histone H3K27ac levels in microglia**

(A) Representative immunofluorescence images showing H3K27ac co-stained with IBA1 in the CA1 region of 5-month-old f/f;AD and cKO;AD mice.

(B) Quantification of H3K27ac intensity in Iba1⁺ microglia from panel (A) (*n* = 4 per group).

Data are presented as mean ± SEM. **p* < 0.05, ***p* < 0.01. Statistical comparisons were made using one-way ANOVA (B) with Tukey’s multiple comparisons test.


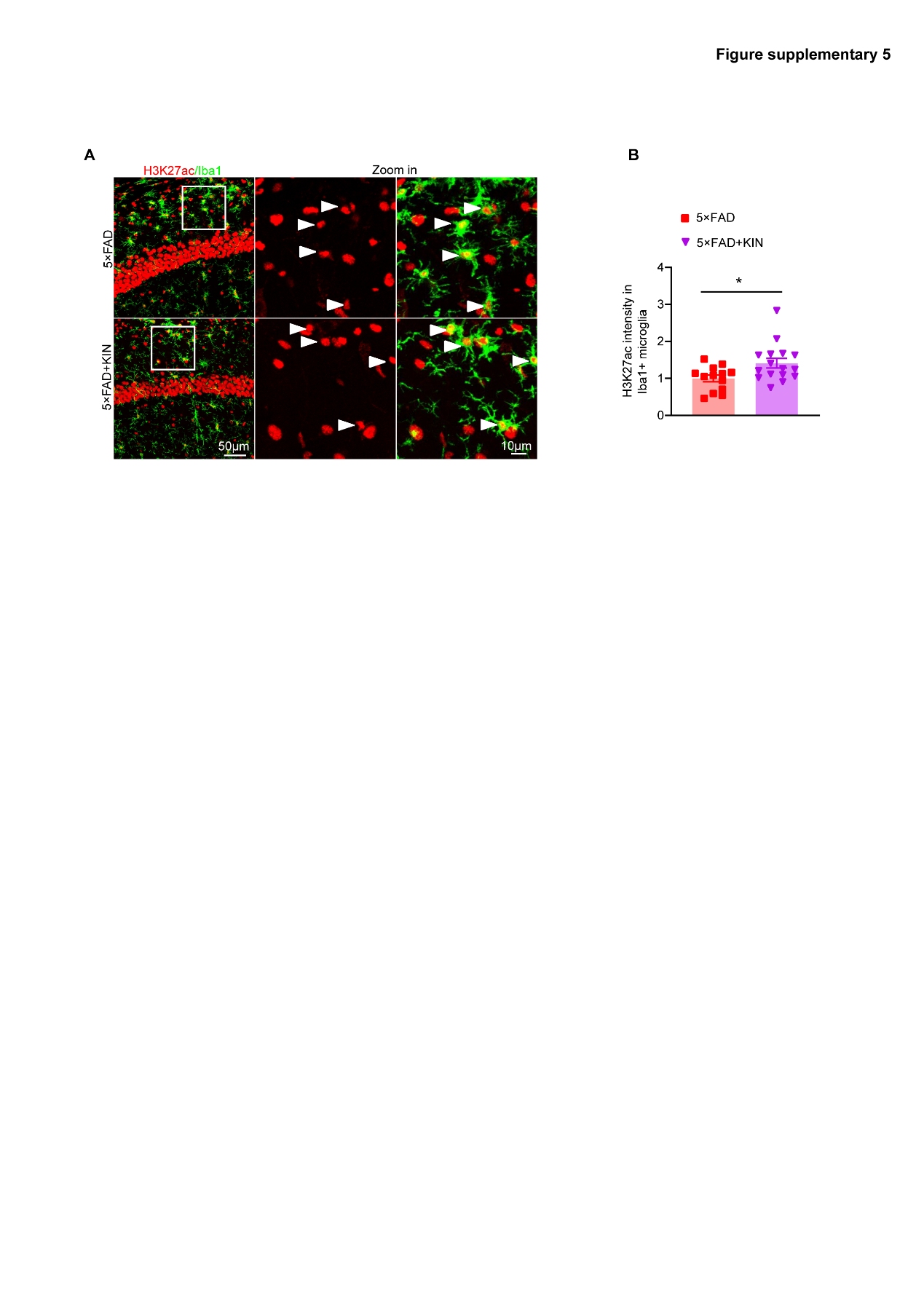


**Figure S6. KIN therapeutic intervention alleviates cognitive decline and AD-** **associated pathology in 5×FAD mice**

(A) The timeline and strategy of KIN treatment in 5×FAD mice. The mice were administered two doses of KIN intragastrically at 25 weeks of age.

(B) The learning curve of escape latency to the platform during the training trials in a Morris water maze test (n = 11 for the saline group and n = 9 for the 50 mg/kg group). (C–F) Memory retention measurements in the probe test of Morris water maze, including mean swimming speed of mice (C), latency to the platform (D), target (platform) entries (E), and time spent in the target quadrant (F).

(G) Representative images of mouse tracks in the probe test.

(H) Representative images of TS staining for Aβ plaques and microglia (Iba1) in brain sections of 5×FAD mice treated with KIN (50 mg/kg) or saline control.

(I–J) Quantification of TS-stained Aβ plaque area (I) and Iba1⁺ microglial immunoreactivity (J) in the dentate gyrus (DG) region.

Data are means ± SEM. *p < 0.05, **p < 0.01, and ***p < 0.001; N.S., not significant. Two-way (B) or one-way ANOVA, followed by Tukey’s multiple comparisons test (C–F) or two-tailed unpaired Student’s t-test (I and J).


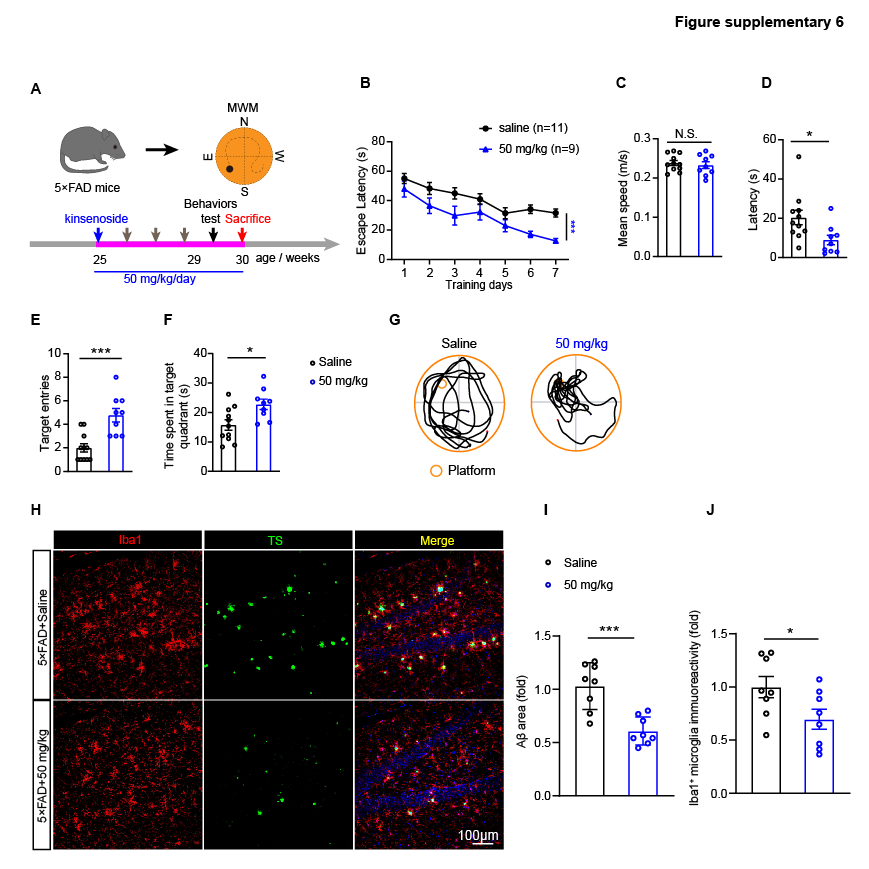


**Figure S7. KIN suppresses neuroinflammation *in vivo* and *in vitro***

(A) Representative images of microglia (Iba1) in the hippocampal DG of 5-month-old f/f;AD and cKO;AD mice (*n* = 4 per group).

(B) Quantification of IBA1⁺ microglia and GFAP⁺ astrocyte immunoreactivity (*n* = 4 per group).

(C–F) qPCR analysis of *Il-6*, *Il-1β*, *Inos* and *Tnf-α* expression in primary microglia treated with KIN or vehicle control after 3 hours of Aβ stimulation (*n* = 3 per group).

Data are presented as mean ± SEM. **p* < 0.05, ****p* < 0.001, *****p* < 0.0001. Statistical comparisons were made using one-tailed unpaired Student’s *t*-test (B) or one-way ANOVA (C-F) with Tukey’s multiple comparisons test.


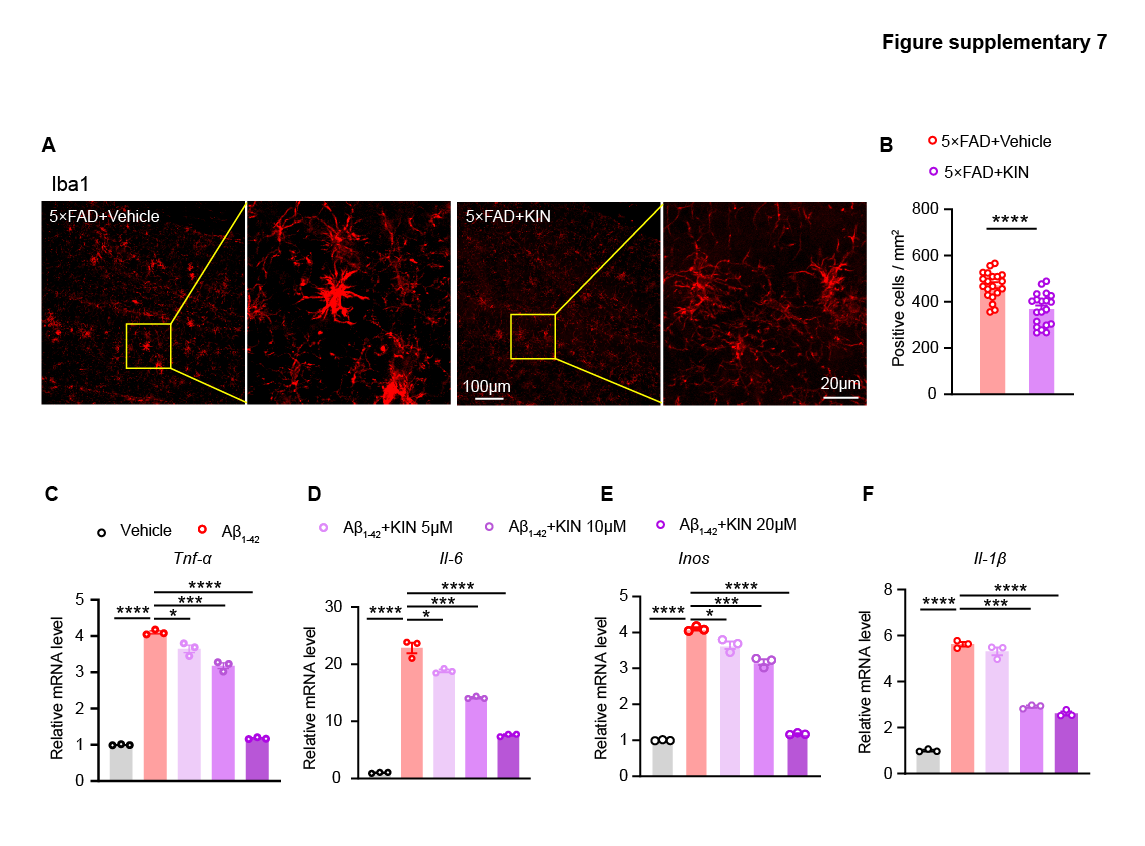


Tables S1–S2 are available in the Supplementary Data.

Table S3. Data collection and refinement statistics

| Statistic | IDH1 PDB: 9M82 |
| --- | --- |
| Data collection |  |
| Beamline | SSRF-BL10U2 |
| Space group | C 1 2 1 |
| Cell dimensions |  |
| a, b, c (Å) | 94.89 112.37 97.76 |
| α, β, γ (°) | 90.00 91.87 90.00 |
| Wavelength (Å) | 0.9791 |
| Chains in ASU | 2 |
| Resolution (Å) | 56.19-3.08 (3.19-3.08) |
| R_merge_ (%) | 0.2109 (0.999) |
| Mean I/σ (I) | 8.40 (1.40) |
| Completeness (%) | 92.41 (82.34) |
| Unique reflection, no. |  |
| Multiplicity | 6.7 (6.9) |
| CC (1/2) | 0.99 (0.633) |
| Refinement |  |
| Resolution (Å) | 56.19-3.08 (3.19-3.08) |
| R_work_/R_free_ (%) | 25.06/30.07 |
| No. of atoms | 6582 |
| Protein | 822 |
| Ligands | 68 |
| B-factor, all atoms (Å) | 63.92 |
| RMS devitationSs |  |
| Bond lengths (Å) | 0.004 |
| Bond angles (°) | 0.83 |
| Ramachandran (%) |  |
| Favored | 92.79 |
| Outliers | 0.61 |

^a^Values in parentheses are for highest-resolution shell. ASU, asymmetric unit; RMS, root mean square.

Table S4. Information on frozen postmortem human frontal lobe samples (related to STAR Methods)

| Case | PMI (h) | Age (years) | Sex | Disease stage |
| --- | --- | --- | --- | --- |
| Control 1 | 21.5 | 78 | Male | Braak 0 |
| Control 2 | 4.3 | 70 | Male | Braak 0 |
| Control 3 | 23.5 | 93 | Male | Braak 0 |
| Control 4 | 4 | 95 | Male | Braak 0 |
| Control 5 | 3 | 89 | Male | Braak 0 |
| AD 1 | 6 | 93 | Male | Braak 1 |
| AD 2 | 9.5 | 94 | Male | Braak 1 |
| AD 3 | 8.8 | 87 | Male | Braak 1 |
| AD 4 | 13.5 | 78 | Male | Braak 1 |
| AD 5 | 4 | 77 | Male | Braak 1 |

Table S5. Primers for plasmid construction

| mutation site | Forward | Reverse |
| --- | --- | --- |
| *D275* | CTGCACGGCACCATCATAGTTTTTACAGGCC | CTGCACGGCACCATCATAGTTTTTACAGGCC |
| *S94* | ATGTGGAAAGGACCAAATGGAACCATACGA | CCATTTGGTCCTTTCCACATTTGTTTCAACT |
| *K212* | TGAGCACCGGAAACACTATTCTGAAGAAATATGAT | ATAGTGTTTCCGGTGCTCAGATACAAAGGC |
| *T77* | CCACTATCGGACCTGATGAGAAGAGGGTT | CTCATCAGGTCCGATAGTGGCACATTTG |
| *N96* | CGGTCTTCGGAGAAGCCATTATCTGCAAA | GGCTTCTCCGAAGACCGTGCCACCCAGAAT |
| *R109* | CGGTCTTCGGAGAAGCCATTATCTGCAAA | ATGGCTTCTCCGAAGACCGTGCCACCCAG |
| *D279* | TGCAGTCGGGCTCTGTGGCCCAAGGGTAT | GCCACAGAGCCCGACTGCACGTCACCATCA |

Table S6. List of qPCR primers, Related to STAR Methods

| Gene | Forward | Reverse |
| --- | --- | --- |
| *m β-actin* | GGCTGTATTCCCCTCCATCG | CCAGTTGGTAACAATGCCATGT |
| *m Il-1β* | TGTAATGAAAGACGGCACACC | TCTTCTTTGGGTATTGCTTGG |
| *m Il-6* | GCTACCAAACTGGATATAATCAGG | CCAGGTAGCTATGGTACTCCAGAA |
| *m Idh1* | ATGCAAGGAGATGAAATGACACG | GCATCACGATTCTCTATGCCTAA |
| *m Tnf-α* | CCCTCACACTCAGATCATCTTCT | GCTACGACGTGGGCTACAG |
| *m Inos* | GTTCTCAGCCCAACAATACAAGA | GTGGACGGGTCGATGTCAC |
| *m Idh2* | GGAGAAGCCGGTAGTGGAGAT | GGTCTGGTCACGGTTTGGAA |
| *m Idh3* | TGGGTGTCCAAGGTCTCTC | TATGCCGCCCACCATACTTAG |
| *h IDH1* | TTGGTGACTTGGTCGTTGGTG | TGTGGTAGAGATGCAAGGAGA |
| *h IDH2* | CGCCACTATGCCGACAAAAG | CCCCATAAGCATGACGACCTAT |
| *h IDH3 β* | GAGCCAAGTCTCAGCGGATT | GGGCATCACAAGCACATCAAA |
| *h IDH3 α* | AATTTCTGGGCCAATACCATCTC | CCCGCGTGGATCTCTAAGG |
| *pET28a-IDH1* | CGGAATTCTCCAAAAAAATCAGTGGCGG | CCGCTCGAGCAAGTTTGGCCTGAGCTAG |
