# Supplementary material for: Kinsenoside Targets IDH1 to Restore Microglial Immune‐Metabolic Homeostasis for Alzheimer's Disease Therapy
Source: Adv Sci (Weinh). 2026 Apr 14;13(36):e75125. doi: 10.1002/advs.75125 (PMC13317708; doi:10.1002/advs.75125)
Supplement: Supplementary file 2 — Supporting File 2: advs75125‐sup‐0002‐Table S1 and S2.docx. [file ADVS-13-e75125-s003.docx]

Table S1. Brain-specific IDH1 co-expressed gene module

| GNAI3 |
| --- |
| PDIA6 |
| KDELR2 |
| FAM96A |
| 2-Sep |
| SSR1 |
| TMCO1 |
| TRIM24 |
| ARF4 |
| HMGN1 |
| DBI |
| CMTM6 |
| LBR |
| CBX3 |
| LYPLA1 |
| DYNLT1 |
| SDCBP |
| PIGF |
| CPNE3 |
| RAB10 |
| SEC11A |
| CYFIP1 |
| TMEM167A |
| ADH5 |
| TCF12 |
| MAPRE1 |
| COPB1 |
| AP3M1 |
| ZFAND6 |
| RP2 |
| TMED10 |
| COPB2 |
| DERA |
| NRAS |
| HIF1A |
| XPOT |
| DRAM2 |
| CCDC90B |
| ABCD3 |
| SEC23B |
| ANXA5 |
| TARS |
| C20ORF72 |
| RPN2 |
| TMEM251 |
| ME2 |
| ABHD3 |
| CCNG1 |
| MTHFD2 |
| SMARCE1 |
| AGPAT5 |
| UGDH |
| C7ORF73 |
| BET1 |
| SLC39A6 |
| PTPRZ1 |
| ARL6IP6 |
| ALG8 |
| MLH1 |
| ALG6 |
| ELOVL5 |
| UBE2E1 |
| ARL5A |
| CDK2AP1 |
| HMGN4 |
| ECI2 |
| AGPS |
| PTTG1IP |
| SRBD1 |
| RIT1 |
| CASP3 |
| PGD |
| YES1 |
| STRADB |
| ASCC3 |
| MIOS |
| HPS5 |
| RSU1 |
| SNX5 |
| HPS3 |
| GALNT1 |
| SPTSSA |
| RDH11 |
| YBX1 |
| CNIH4 |
| RALA |
| RPS27L |
| ACSL3 |
| PGM3 |
| PRRC1 |
| DESI2 |
| LIMA1 |
| C2ORF43 |
| OSBPL9 |
| TMEM60 |
| MSMO1 |
| PON2 |
| ZNF22 |
| TMEM218 |
| PTPN13 |
| CCDC50 |
| TMEM69 |
| LAPTM4B |
| ACOX1 |
| IVNS1ABP |
| LEPROTL1 |
| USP3 |
| ACTG1 |
| COQ2 |
| ROBO1 |
| TSPAN6 |
| NUPL2 |
| MARCKS |
| SGPL1 |
| TBCCD1 |
| CYP20A1 |
| BCHE |
| RBM8A |
| WDR41 |
| PYGL |
| CYP51A1 |
| IGF2BP3 |
| DCAF12 |
| TXNDC5 |
| CASK |
| GOLM1 |
| MEST |
| TRIB2 |
| FAM70A |
| PSPH |
| CRISPLD1 |
| ZNF300 |
| FABP7 |
| SALL1 |
| LINC00493 |
| SNHG6 |
| ZNF605 |
| ZEB1-AS1 |
| LOC643072 |
| LOC90784 |
| PAN3-AS1 |
| FAM228B |
| FAM217B |
| TICAM2 |
| SNHG9 |
| AK4 |
| CEP85L |
| LOC100287015 |
| LOC100132707 |
| LOC100129195 |
| LINC00294 |
| LOC645212 |
| LOC150381 |
| ILDR2 |
| FAM66C |
| TMPO-AS1 |
| HTATSF1P2 |
| LPP-AS2 |
| A2M-AS1 |
| LOC145474 |
| STEAP1B |
| SNORA72 |
| GBP1P1 |
| LOC401321 |
| LOC339535 |
| CHL1-AS2 |
| SHISA6 |
| HCG11 |
| LOC100288152 |
| LOC100270804 |
| LOC255512 |
| SNHG4 |
| C6ORF147 |
| SBF2-AS1 |
| LOC100144603 |
| LOC100289187 |
| ANKRD18A |
| ZC3H12D |
| TBC1D30 |
| LOC401068 |
| C10ORF112 |
| LINC00323 |
| HMGB3P30 |
| LOC730236 |
| LINC00271 |
| PVALB |
| DCTN1-AS1 |
| RFPL2 |
| RSPO2 |
| CYP11A1 |
| SAMD12 |
| KLHL10 |
| PNLDC1 |
| HPCAL4 |
| TNNI3K |
| C4ORF45 |
| NRG4 |
| GFOD1 |
| TMEM155 |
| OXGR1 |
| MTUS2 |
| DGKE |
| FAM163A |
| WFDC10A |
| RHOXF1 |
| ZFP57 |
| PHACTR1 |
| ADARB1 |
| SNX32 |
| PABPC1L2B |
| TNNT2 |
| SMPX |
| KCNQ5 |
| LINGO2 |
| ZBTB7C |
| MFSD4 |
| KCTD1 |
| KCNV1 |
| FBXO27 |
| TRABD2A |
| C19ORF77 |
| FAM81A |
| FBXL16 |
| MBL1P |
| KIAA1324 |
| PPM1J |
| GLS2 |
| USP2 |
| LHX6 |
| NPTX1 |
| RTN4RL1 |
| SGPP2 |
| RNF43 |
| CHRM4 |
| UNC5A |
| CAMKK2 |
| FRMPD4 |
| TEX29 |
| RTP1 |
| PNMT |
| GRIN2C |
| SLC32A1 |
| C1ORF204 |
| ATG9B |
| KRT81 |
| KRT17 |
| TSR2 |
| HSPB3 |
| RHEBL1 |
| PPP4R4 |
| PRRT3 |
| BEGAIN |
| KDM8 |
| RXFP1 |
| SH3RF2 |
| WBSCR17 |
| SVOP |
| VPS53 |
| SH2D4B |
| ASB2 |
| STX1B |
| RHOF |
| NGEF |
| BCAS4 |
| PCBP3 |
| BRSK2 |
| GLP2R |
| ANKRD34A |
| FGF18 |
| ROS1 |
| CHRM1 |
| CYP4A11 |
| PITPNM3 |
| CAMK4 |
| UPB1 |
| RHOBTB2 |
| TSSK1B |
| HCN1 |
| CA11 |
| FLJ23867 |
| CRHR2 |
| FXYD7 |
| F7 |
| C15ORF27 |
| OTUD7A |
| FUT1 |
| KCNC2 |
| HAS1 |
| CHRNA6 |
| GPR68 |
| LOC100130100 |
| KCNA4 |
| PRSS3P2 |
| UBOX5 |
| LOC339524 |
| ADRA1B |
| OSBP2 |
| PADI3 |
| C10ORF35 |
| TRIM29 |
| CLDND2 |
| PART1 |
| HTR6 |
| PPP1R1B |
| KCNQ4 |
| FAAH |
| MIR600HG |
| ALDOB |
| KY |
| RAB3A |
| LOC389332 |
| FAM135B |
| KSR2 |
| RAB11FIP4 |
| PITPNM1 |
| KCNK3 |
| SH3PXD2A |
| NRAP |
| SYNGR1 |
| HIPK4 |
| PYGM |
| TRIM17 |
| DRD5 |
| BAIAP2L2 |
| MGAT5B |
| ANK1 |
| RELL2 |
| COLQ |
| ITPKA |
| WSCD2 |
| DRD1 |
| GPR21 |
| KCNH1 |
| CITED4 |
| LRRC73 |
| PAX2 |
| PPP1R9B |
| CD22 |
| KCNC3 |
| FATE1 |
| RS1 |
| KRT31 |
| SLIT3 |
| PRRG3 |
| GAD2 |
| KCNAB2 |
| SMYD1 |
| LOC730098 |
| DTNB |
| AGAP2 |
| MOB2 |
| CACNG3 |
| SSTR4 |
| PSD |
| OTUD5 |
| LRRC43 |
| SLC6A13 |
| CORO6 |
| HTR1E |
| ALOX12B |
| GIT1 |
| PRKACG |
| KIAA1045 |
| ZNF483 |
| CCDC13 |
| SCN1B |
| GJD2 |
| PTK2B |
| MUC6 |
| TRPV3 |
| CLSTN3 |
| 12-Sep |
| TNNC2 |
| C21ORF67 |
| EPHB6 |
| DNAH6 |
| VAMP2 |
| CPLX2 |
| MAPRE3 |
| IRF2BP1 |
| PLAC2 |
| KCNS1 |
| GOLGA7B |
| EGR4 |
| SLC25A42 |
| ANKRD2 |
| TCTE1 |
| LY6D |
| GPR61 |
| PLCH2 |
| LRFN2 |
| FBXO44 |
| IL17C |
| IL1RL2 |
| MAPK3 |
| SNCB |
| MASP2 |
| PAX7 |
| C12ORF54 |
| TNFRSF25 |
| KNCN |
| HTR3B |
| CALY |
| TOM1L2 |
| SYT12 |
| SH3GLB2 |
| ICOSLG |
| MINK1 |
| MEPE |
| PELI3 |
| ADPRHL1 |
| PRRT2 |
| KIFC2 |
| IGFALS |
| CHRNA2 |
| BZRAP1 |
| ST6GALNAC6 |
| CCNO |
| RAB11FIP5 |
| SCRT1 |
| TUBA8 |
| BRSK1 |
| 5-Sep |
| MAP2K7 |
| YPEL4 |
| MGAT3 |
| NR1D1 |
| CAMK1G |
| HPCA |
| HAR1A |
| CACNA1B |
| IGHMBP2 |
| MATK |
| YJEFN3 |
| ZNF335 |
| TESPA1 |
| RBFOX3 |
| CHD5 |
| OPN4 |
| C6ORF106 |
| MYO15A |
| MADCAM1 |
| DKFZP434A062 |
| SOHLH1 |
| HR |
| CPNE9 |
| IHH |
| LGI3 |
| LRRC56 |
| NOS1 |
| PPP1R37 |
| OPRL1 |
| LOC728743 |
| KCNJ4 |
| PLK5 |
| KCNA1 |
| EPHA8 |
| LOC80054 |
| SYN2 |
| MAPK15 |
| PCDHGC5 |
| MESP2 |
| MYOZ3 |
| GLP1R |
| PLIN4 |
| DHRS7C |
| PIN1P1 |
| NCR2 |
| DOC2A |
| HDAC11 |
| STX1A |
| ICAM5 |
| GRM4 |
| GRIN2A |
| PVRL1 |
| PKD1 |
| PACSIN1 |
| SYP |
| PARD6A |
| GPR135 |
| CEND1 |
| IL5RA |
| ARHGDIG |
| KCNT1 |
| MBLAC1 |
| ALS2CL |
| PITPNM2 |
| MUC3A |
| RASD2 |
| CPNE6 |
| SLC9A5 |
| NGB |
| PNCK |
| ABCC12 |
| CD5 |
| SYNGAP1 |
| SLC8A2 |
| LOC100128292 |
| ABCB9 |
| GFRA4 |
| KRT83 |
| NCS1 |
| RBFOX1 |
| SPRN |
| KLK7 |
| MAP3K9 |
| BAIAP3 |
| SHANK1 |
| ABCG4 |
| CALHM1 |
| CYGB |
| SLC16A11 |
| DYRK1B |
| GRM1 |
| PICK1 |
| DLGAP3 |
| DUSP8 |
| ADRB3 |
| TMEM59L |
| IQSEC3 |
| MCHR2 |
| MRVI1-AS1 |
| BTBD9 |
| GRM2 |
| ATP4A |
| DNAH1 |
| RASGRF1 |
| SFTPD |
| CACNA1F |
| NOXA1 |
| DDN |
| INSL3 |
| TACR2 |
| TSPYL2 |
| RHO |
| TRIM3 |
| PDE4A |
| CCKBR |
| SEMA6B |
| MPL |
| LYNX1 |
| MYH14 |
| IL34 |
| TRMT61A |
| KCNB2 |
| C1ORF222 |
| NCDN |
| TBC1D25 |
| KIAA1751 |
| CHRD |
| ADAMTS8 |
| CACNA1C |
| GFRA2 |
| KCTD17 |
| CLEC2L |
| LRTM2 |
| KCNC4 |
| PDK2 |
| RUSC2 |
| PHYHIP |
| CNGB1 |
| ALOXE3 |
| SGSM1 |
| KCNJ12 |
| SYN1 |
| KLK5 |
| MAPK11 |
| CRY2 |
| CIDEA |
| MAPK8IP2 |
| CD6 |
| HTR5A |
| KLC2 |
| CPNE7 |
| CPLX3 |
| PRRT1 |
| FKBP8 |
| CDH22 |
| SPEF1 |
| GALNT9 |
| SPDEF |
| MYH7B |
| ZFYVE28 |
| SLC30A3 |
| SSTR3 |
| PNMAL2 |
| GAS2L2 |
| INPP5J |
| ABHD8 |
| ATXN7L3 |
| NPM2 |
| SFTPC |
| STAC2 |
| REXO1 |
| ASPDH |
| FBXO40 |
| RASAL1 |
| KCNS2 |
| BAIAP2 |
| EXTL1 |
| ABTB1 |
| KCNIP3 |
| C2CD2L |
| PANX2 |
| WNK2 |
| RASSF7 |
| PRSS3 |
| PEX16 |
| SLC6A7 |
| VWA3A |
| CACNA1I |
| KCNJ3 |
| RLTPR |
| TEF |
| VWA5B2 |
| CABP1 |
| PRMT8 |
| SYT5 |
| VIPR1 |
| PDE4C |
| FSTL4 |
| NAT6 |
| EMX1 |
| B3GNT4 |
| GNG13 |
| NEURL |
| GRASP |
| PLEKHG5 |
| PDZD7 |
| CACNG2 |
| WNT10B |
| ABLIM2 |
| IQSEC2 |
| CA7 |
| C17ORF28 |
| CNNM1 |
| ANKRD24 |
| EPB49 |
| SH2D5 |
| GABRD |
| SLC7A4 |
| SNCG |
| SYT7 |
| HTR4 |
| CRHR1 |
| KCNJ9 |
| C21ORF2 |
| EPHA10 |
| CA4 |
| ATP2B3 |
| CRTC1 |
| PDE1B |
| MAP3K10 |
| HRH3 |
| KCNH3 |
| MAPK8IP3 |
| SCN2B |
| PNMA3 |
| ZER1 |
| KCNN1 |
| PNPLA7 |
| ZBTB7A |
| CAMKK1 |
| KNDC1 |
| SPTB |
| SPRYD3 |
| KIF17 |
| PPP1R3F |
| SPTBN4 |
| CACNB1 |
| SLC6A17 |
| PRKCG |
| ADAM11 |
| GRIN1 |
| MEF2D |

Table S2. Effect size (ES) and FDR of BIM expression differences between AD and non-AD in hippocampus, temporal cortex, and frontal cortex.

| Phenotype | ES | FDR |
| --- | --- | --- |
| AD vs. non-AD in hippocampus | -0.24803 | 0 |
| AD vs. non-AD in temporal cortex | -0.24458 | 0 |
| AD vs. non-AD in frontal cortex | -0.19774 | 0 |
