# Supplementary figures and images for: Kinsenoside Targets IDH1 to Restore Microglial Immune‐Metabolic Homeostasis for Alzheimer's Disease Therapy
Source: Adv Sci (Weinh). 2026 Apr 14;13(36):e75125. doi: 10.1002/advs.75125 (PMC13317708; doi:10.1002/advs.75125)

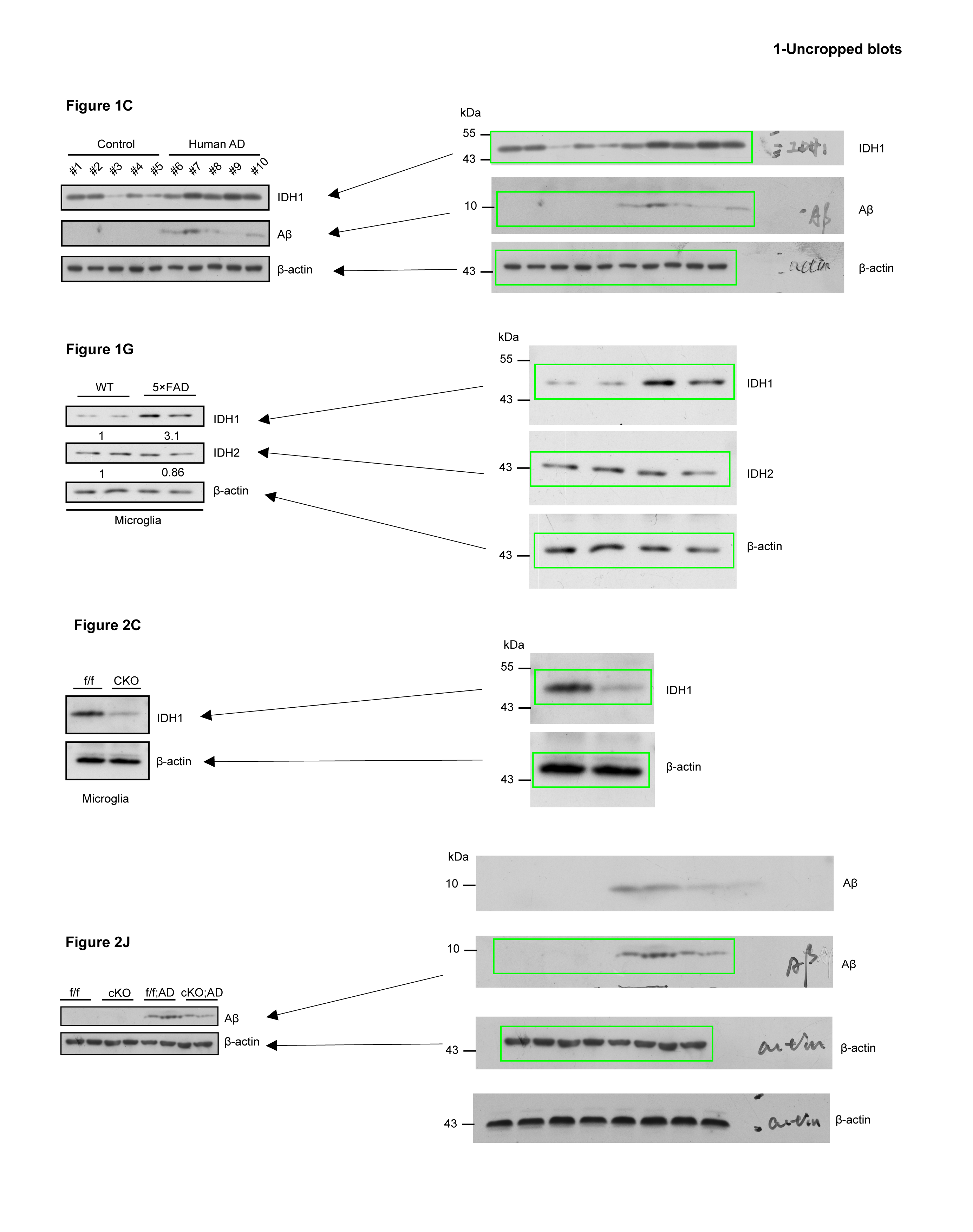

Supplement: Supplementary file 3 — Supporting File 3: advs75125‐sup‐0003‐Uncropped blots.zip. [file ADVS-13-e75125-s001.zip › 1-Uncropped blots-01.tif]

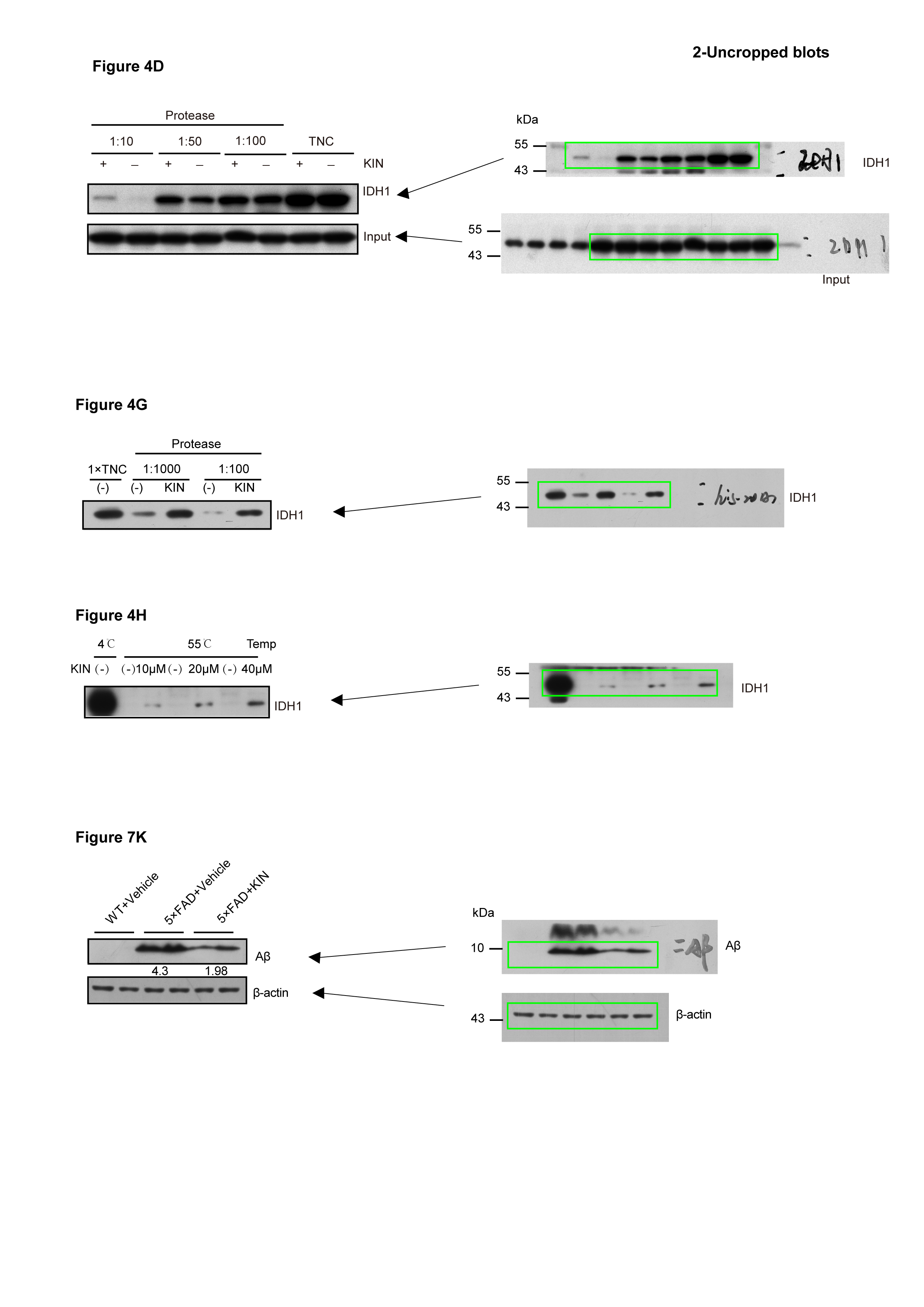

Supplement: Supplementary file 3 — Supporting File 3: advs75125‐sup‐0003‐Uncropped blots.zip. [file ADVS-13-e75125-s001.zip › 2-Uncropped blots-01.tif]
